# Supplementary material for: Identification of the co-regulatory siRNAs of “miRNA→target” in Oryza sativa
Source: PLoS One. 2025 Apr 3;20(4):e0321182. doi: 10.1371/journal.pone.0321182 (PMC11967944; doi:10.1371/journal.pone.0321182)
Supplement: S1 Fig — All 5 degradome sequencing data libraries (GSM434596, GSM455938, GSM4113551, GSM455939, GSM476257) were recruited for T-plot profiling. The IDs of the target transcripts and the corresponding miRNAs are listed on the top of sub-figures. The x axes measure the positions of the degradome signals along the transcripts, and the y axes measure the degradome signal intensities based on normalized counts (in RPM, reads per million).The binding sites of the miRNA on their target transcripts were denoted by red horizontal lines. No specific cleavage signals have been found in the middle of the miRNA binding sites in these target genes. (PDF) [file pone.0321182.s001.pdf]

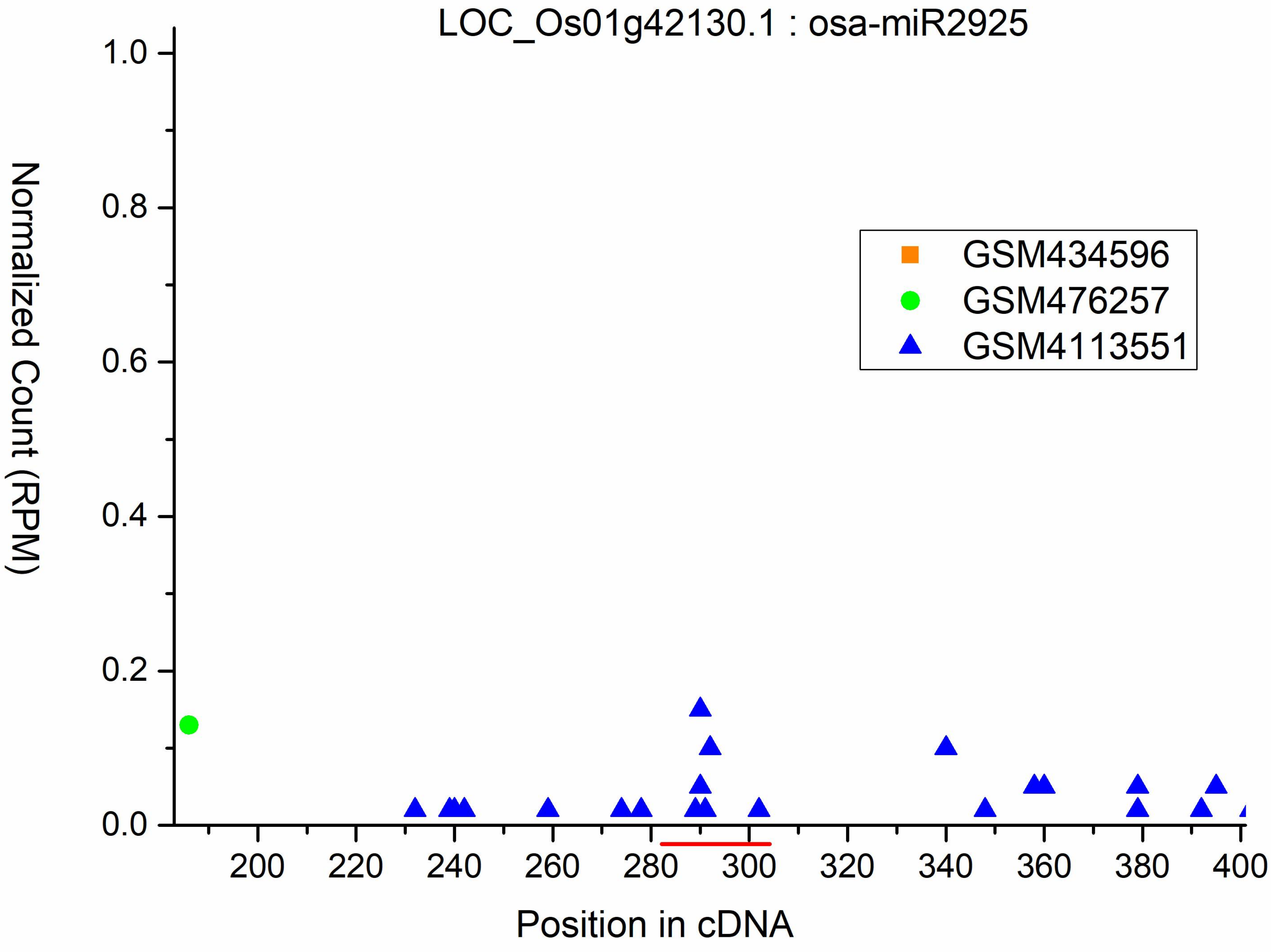

LOC_Os01g42130.1 : osa-miR2925
Normalized Count (RPM)
1.0
0.8
0.6
0.4
0.2
0.0
200
220
240
260
280
300
320
340
360
380
400
Position in cDNA
GSM434596
GSM476257
GSM4113551

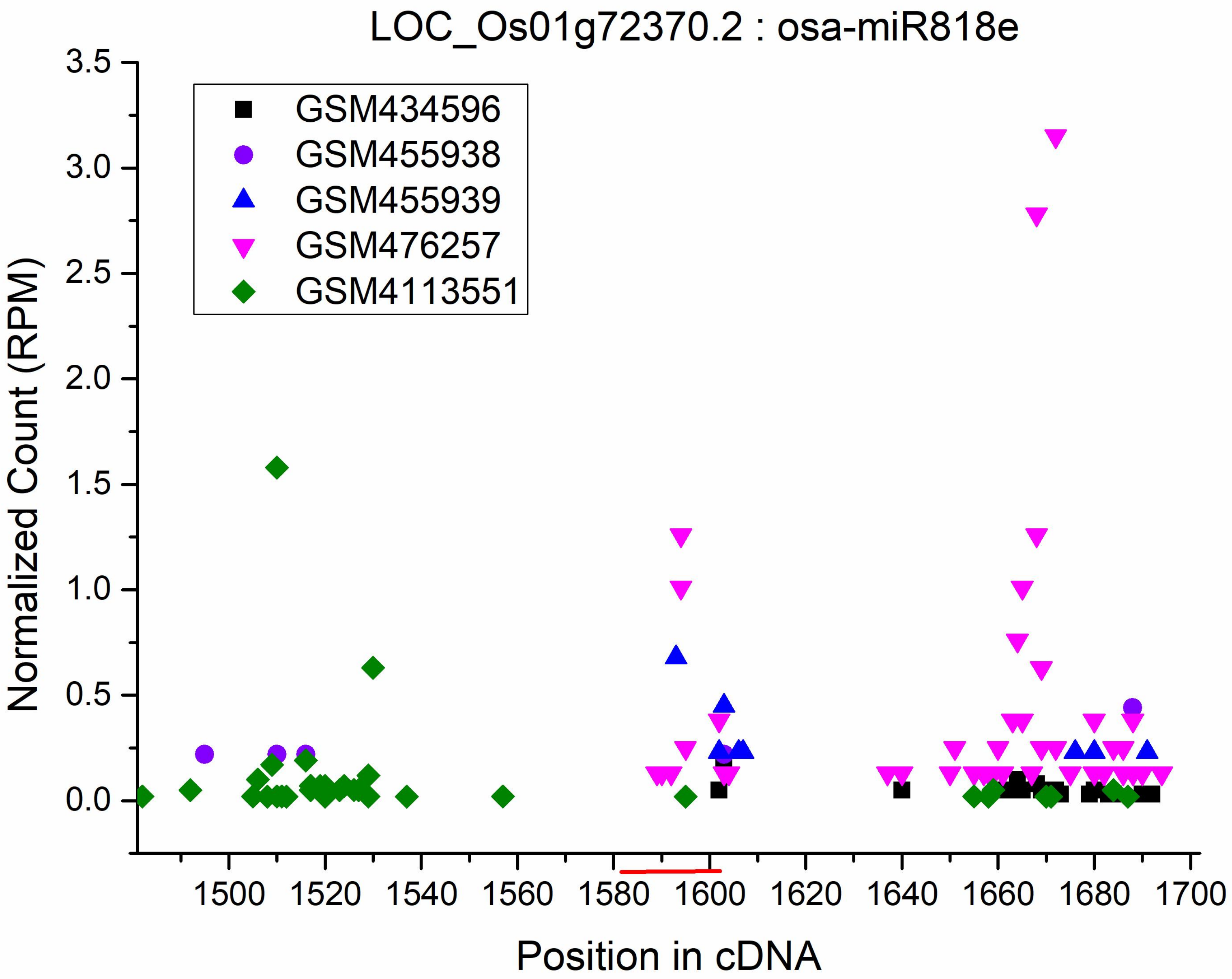
LOC_Os01g72370.2 : osa-miR818e
GSM434596
GSM455938
GSM455939
GSM476257
GSM4113551
Normalized Count (RPM)
3.5
3.0
2.5
2.0
1.5
1.0
0.5
0.0
1500
1520
1540
1560
1580
1600
1620
1640
1660
1680
1700
Position in cDNA

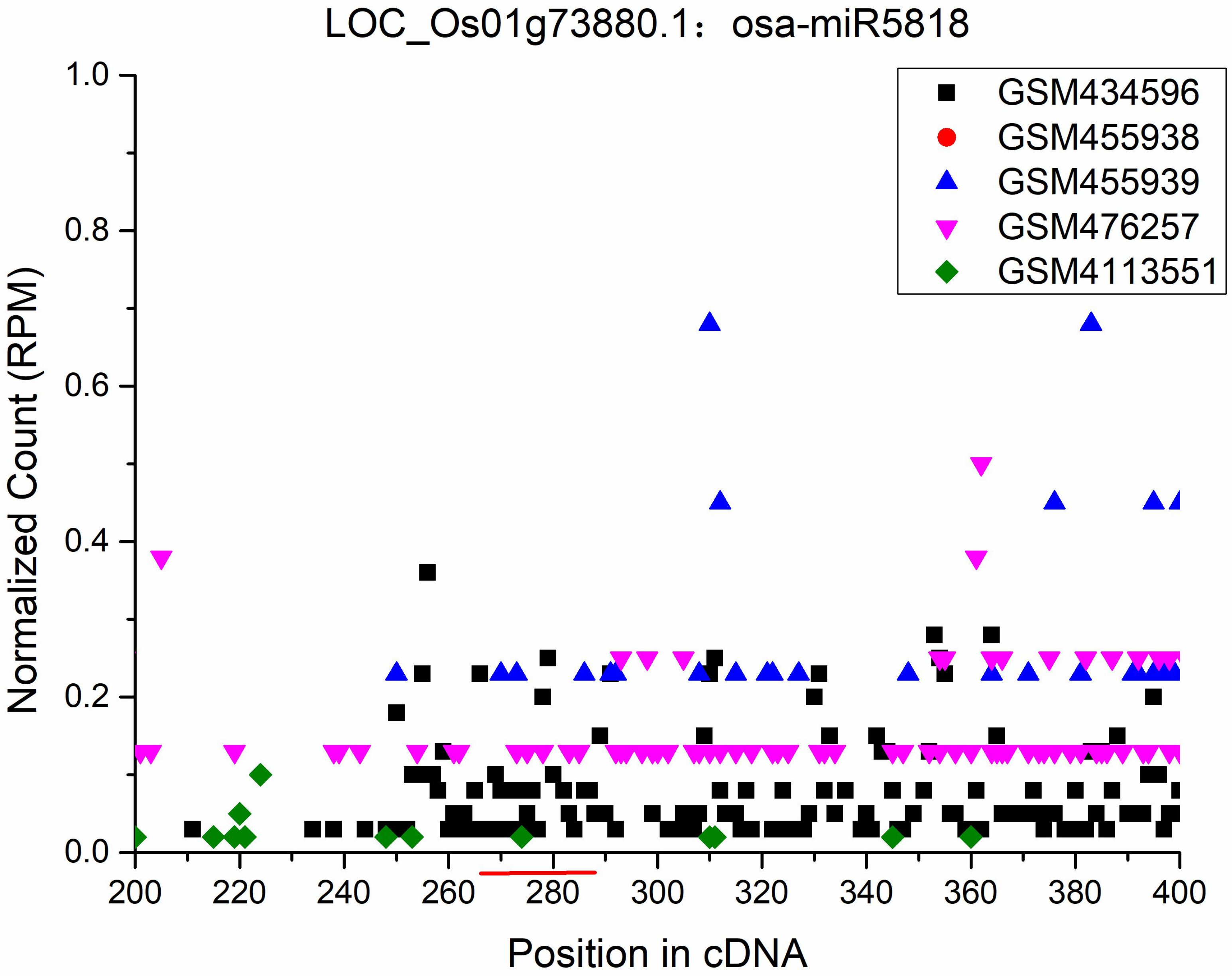

LOC_Os01g73880.1：osa-miR5818
Normalized Count (RPM)
1.0
0.8
0.6
0.4
0.2
0.0
200
220
240
260
280
300
320
340
360
380
400
Position in cDNA
GSM434596
GSM455938
GSM455939
GSM476257
GSM4113551

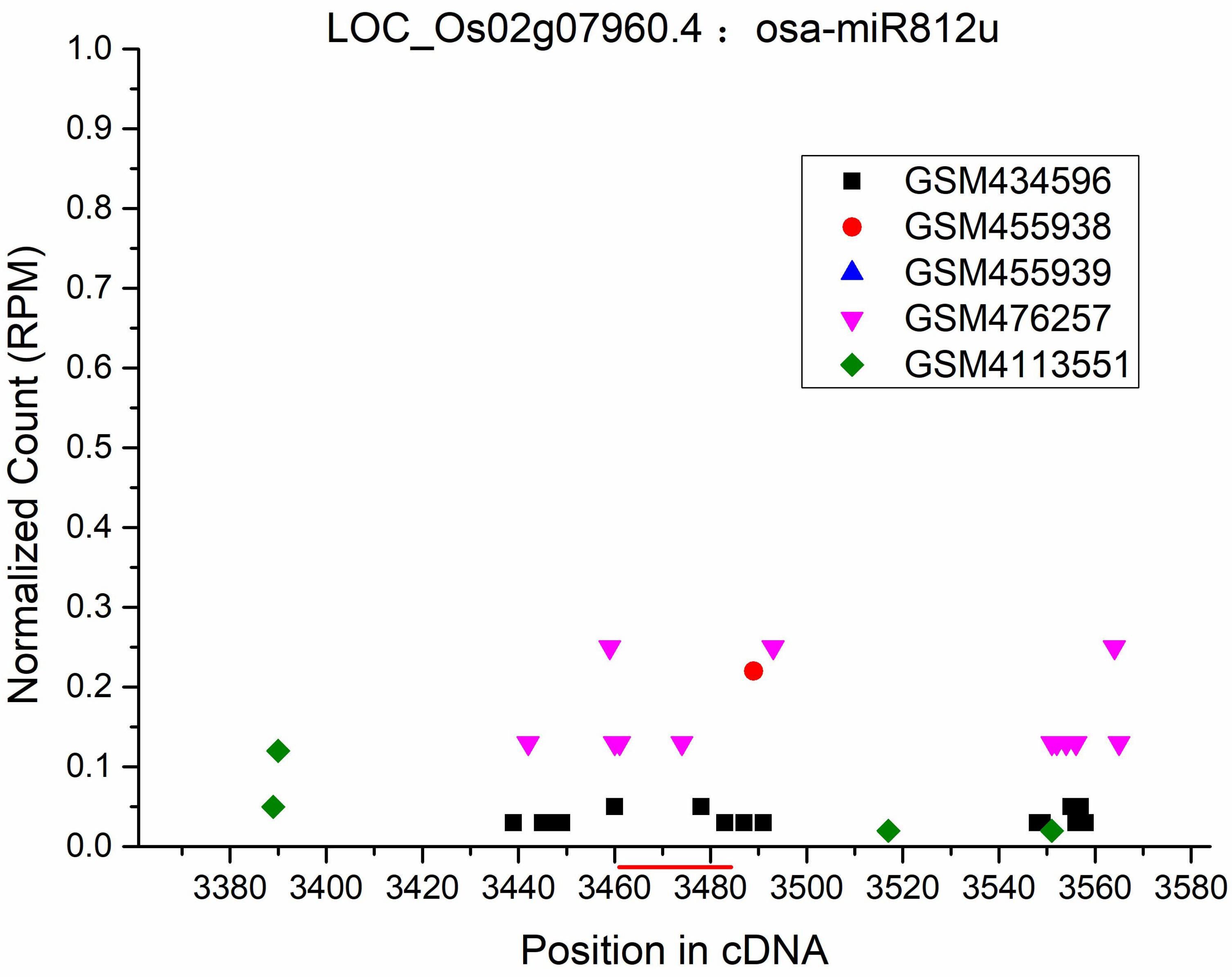
LOC_Os02g07960.4 ： osa-miR812u
Normalized Count (RPM)
1.0
0.9
0.8
0.7
0.6
0.5
0.4
0.3
0.2
0.1
0.0
3380
3400
3420
3440
3460
3480
3500
3520
3540
3560
3580
Position in cDNA
GSM434596
GSM455938
GSM455939
GSM476257
GSM4113551

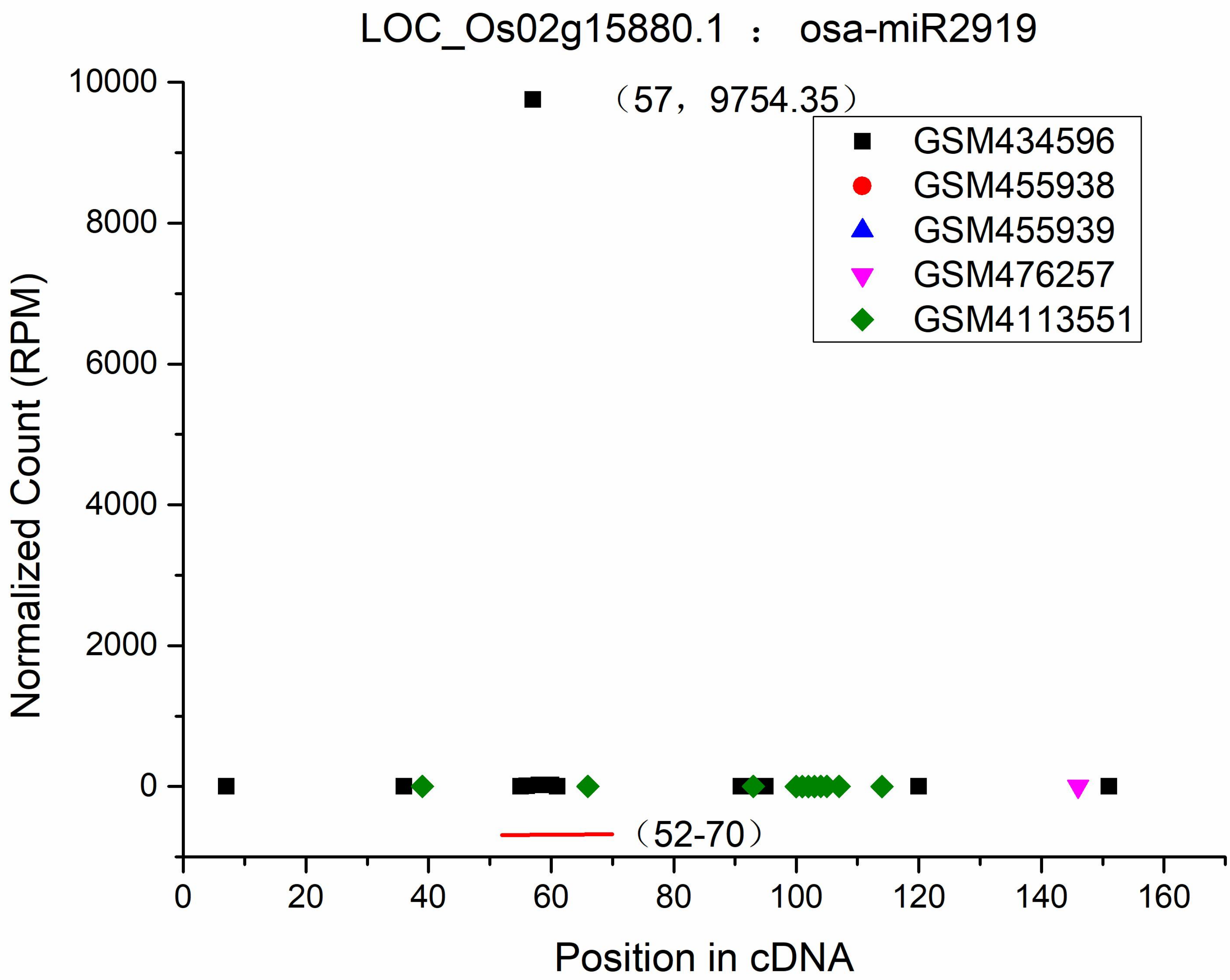
LOC_Os02g15880.1 ： osa-miR2919
（57，9754.35）
GSM434596
GSM455938
GSM455939
GSM476257
GSM4113551
（52-70）
Normalized Count (RPM)
10000
8000
6000
4000
2000
0
0
20
40
60
80
100
120
140
160
Position in cDNA

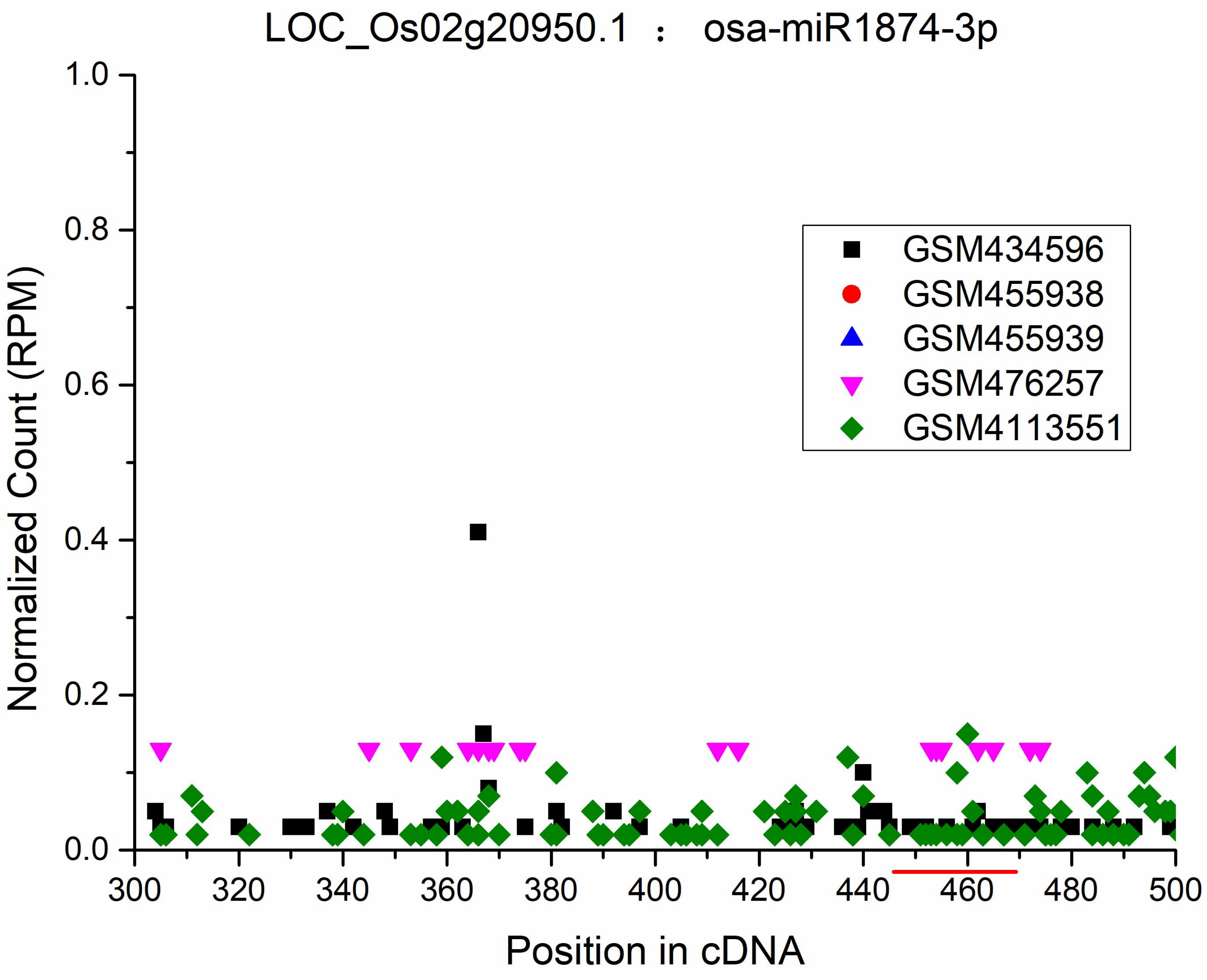

LOC_Os02g20950.1 ： osa-miR1874-3p
Normalized Count (RPM)
0.0
0.2
0.4
0.6
0.8
1.0
Position in cDNA
300
320
340
360
380
400
420
440
460
480
500
GSM434596
GSM455938
GSM455939
GSM476257
GSM4113551

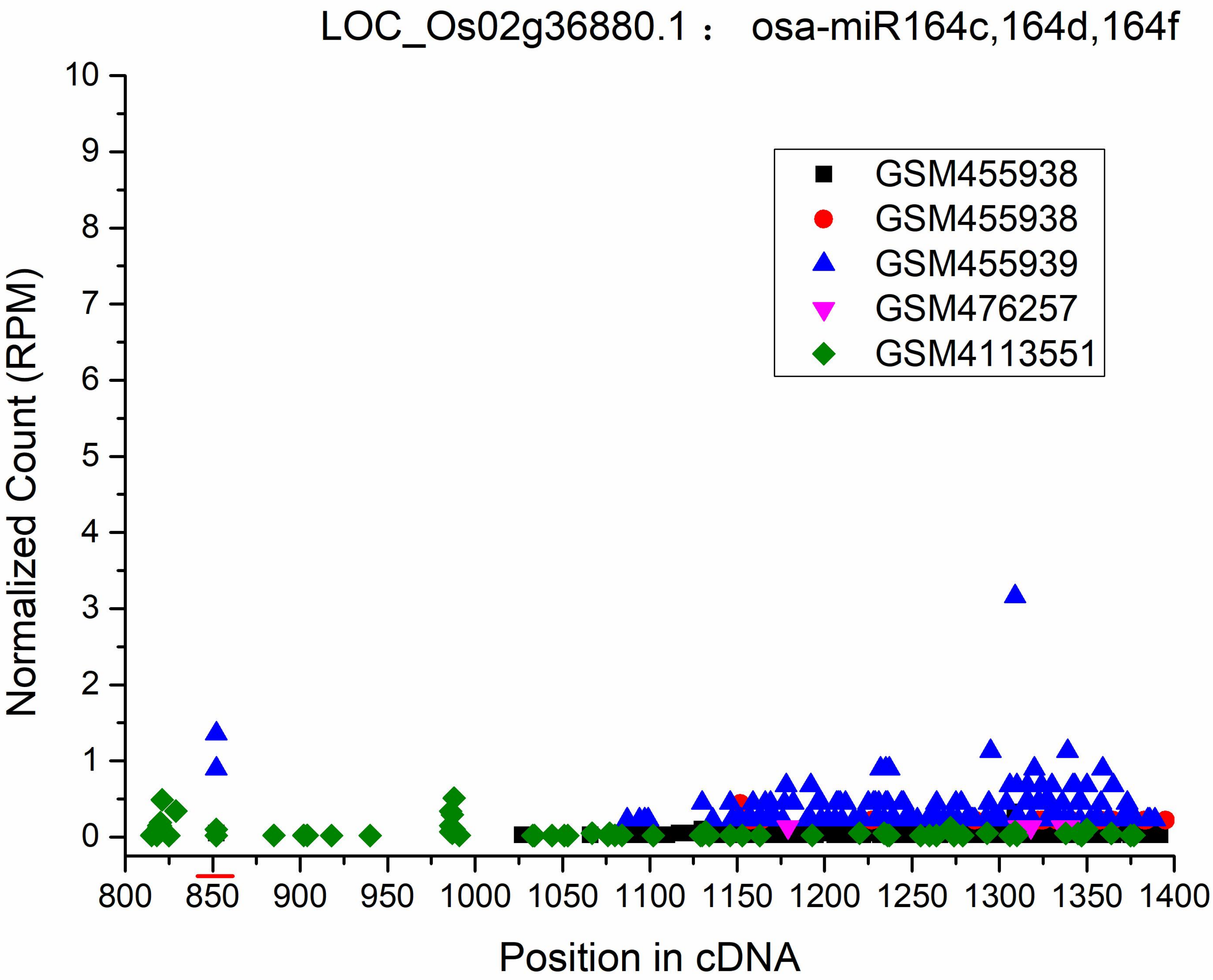

LOC_Os02g36880.1： osa-miR164c,164d,164f
Normalized Count (RPM)
Position in cDNA
GSM455938
GSM455938
GSM455939
GSM476257
GSM4113551
0
1
2
3
4
5
6
7
8
9
10
800
850
900
950
1000
1050
1100
1150
1200
1250
1300
1350
1400

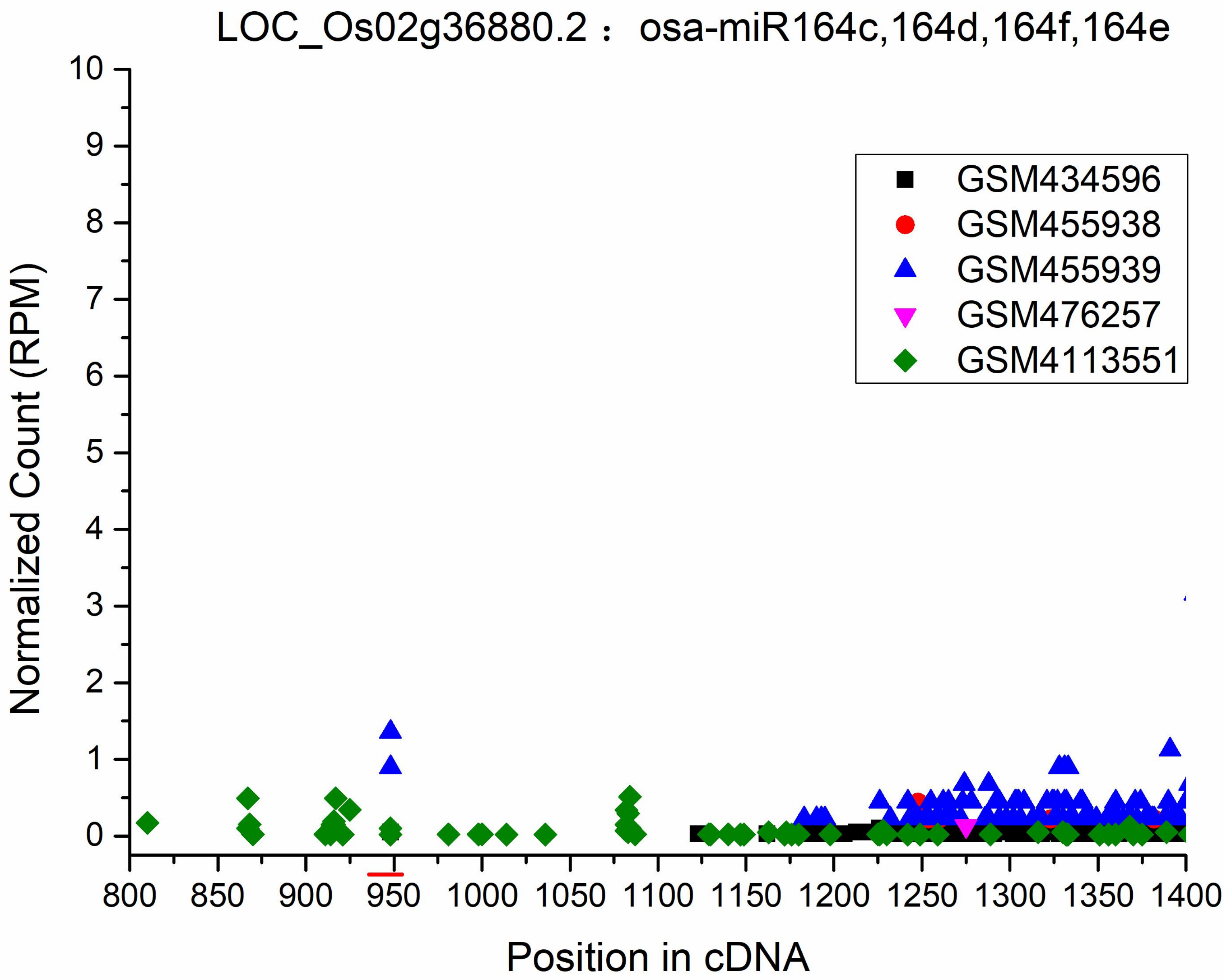

LOC_Os02g36880.2 ： osa-miR164c,164d,164f,164e
Normalized Count (RPM)
0
1
2
3
4
5
6
7
8
9
10
800
850
900
950
1000
1050
1100
1150
1200
1250
1300
1350
1400
Position in cDNA
GSM434596
GSM455938
GSM455939
GSM476257
GSM4113551

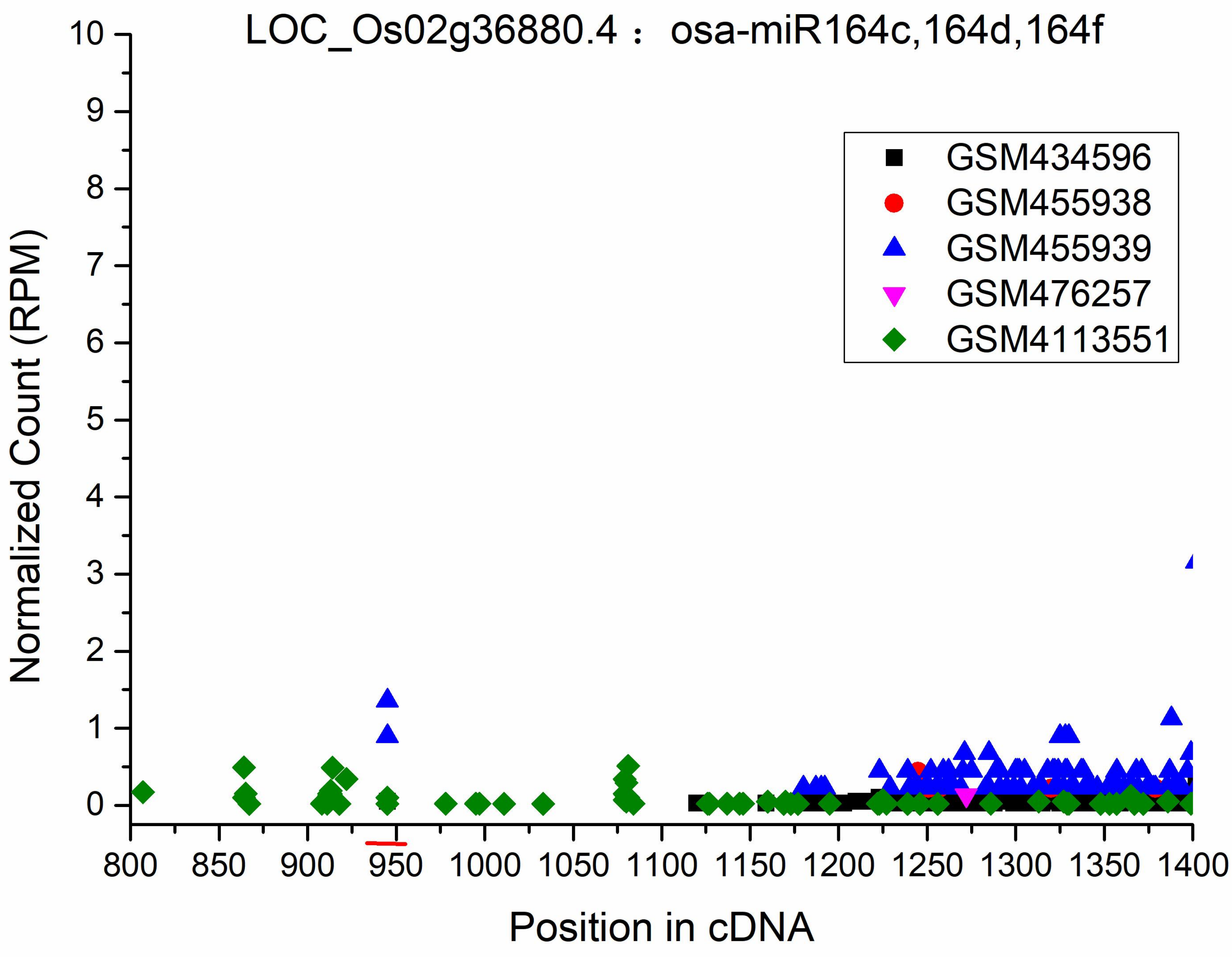

LOC_Os02g36880.4： osa-miR164c,164d,164f
Normalized Count (RPM)
Position in cDNA
GSM434596
GSM455938
GSM455939
GSM476257
GSM4113551
0
1
2
3
4
5
6
7
8
9
10
800
850
900
950
1000
1050
1100
1150
1200
1250
1300
1350
1400

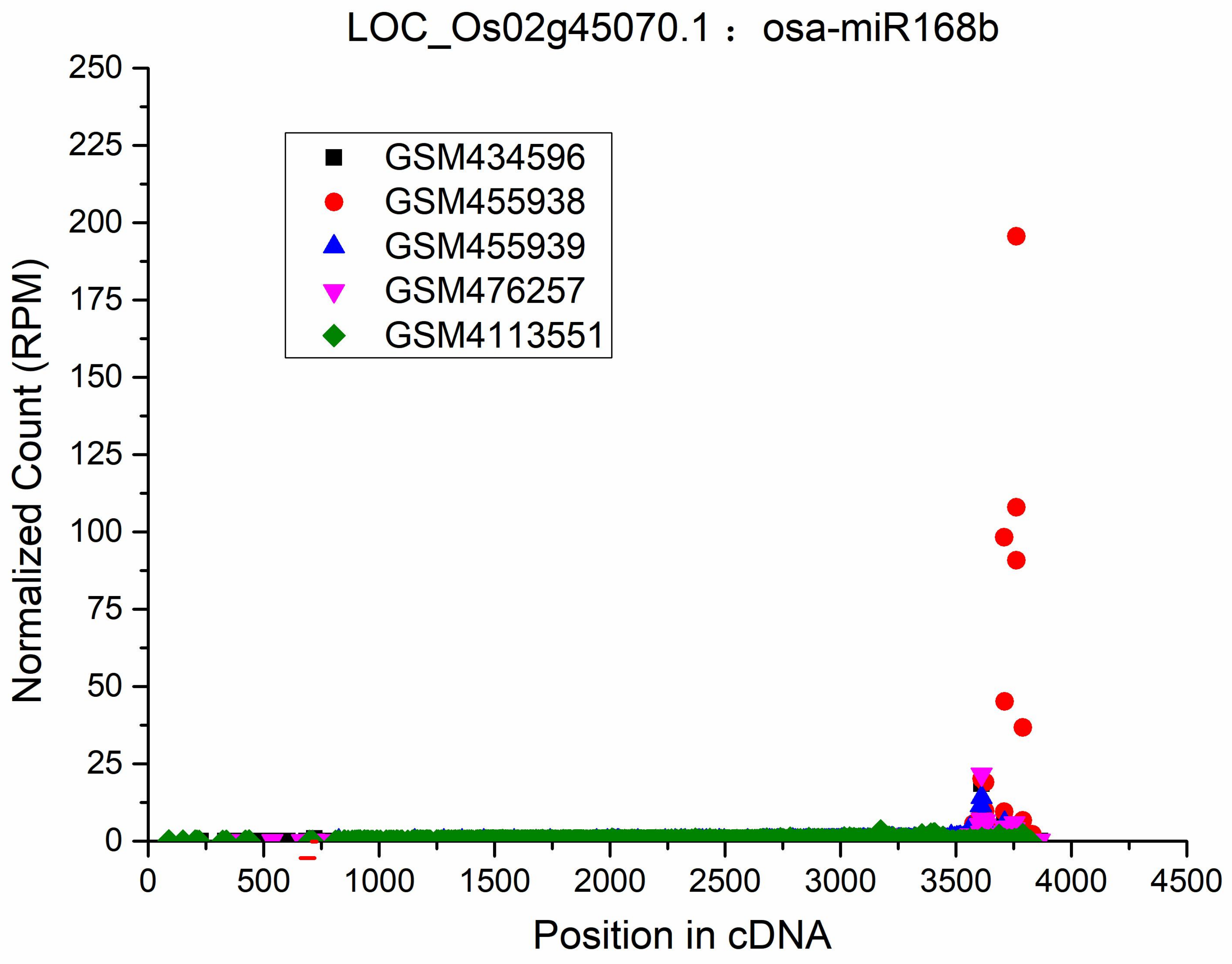
LOC_Os02g45070.1 ： osa-miR168b
Normalized Count (RPM)
Position in cDNA
GSM434596
GSM455938
GSM455939
GSM476257
GSM4113551
0
25
50
75
100
125
150
175
200
225
250
0
500
1000
1500
2000
2500
3000
3500
4000
4500

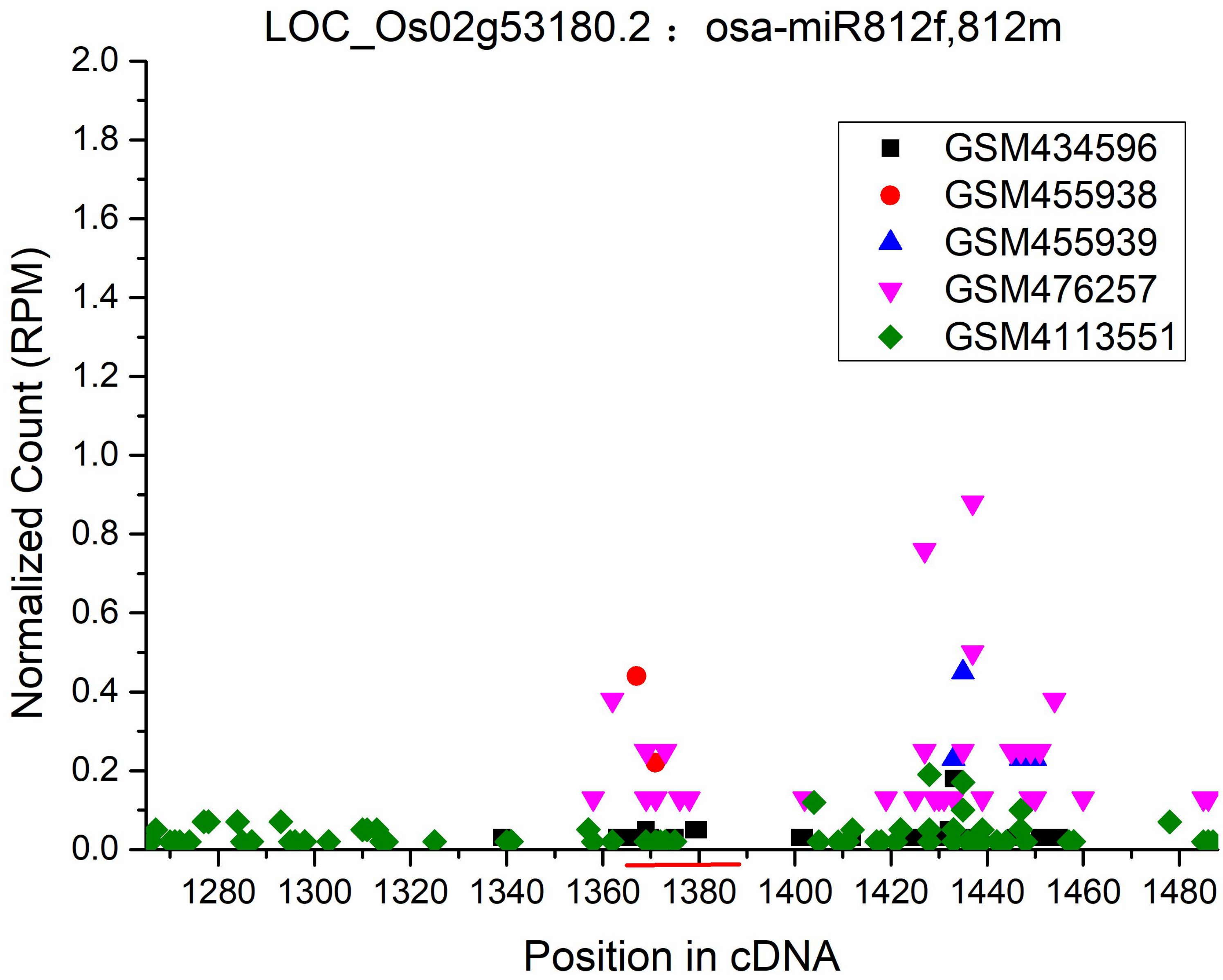
LOC_Os02g53180.2 ： osa-miR812f,812m
Normalized Count (RPM)
2.0
1.8
1.6
1.4
1.2
1.0
0.8
0.6
0.4
0.2
0.0
1280
1300
1320
1340
1360
1380
1400
1420
1440
1460
1480
Position in cDNA
GSM434596
GSM455938
GSM455939
GSM476257
GSM4113551

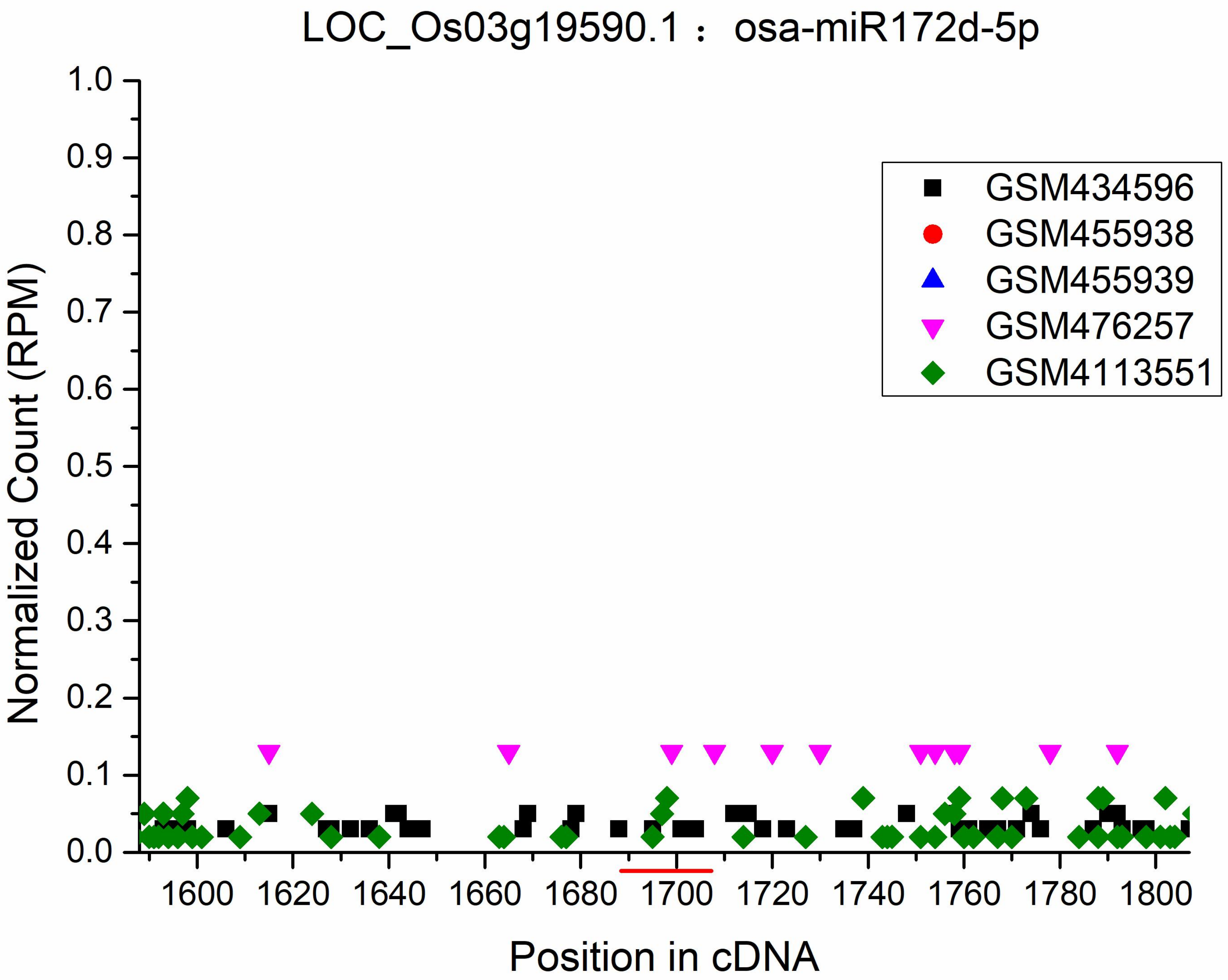

LOC_Os03g19590.1 ： osa-miR172d-5p
Normalized Count (RPM)
1.0
0.9
0.8
0.7
0.6
0.5
0.4
0.3
0.2
0.1
0.0
1600
1620
1640
1660
1680
1700
1720
1740
1760
1780
1800
Position in cDNA
GSM434596
GSM455938
GSM455939
GSM476257
GSM4113551

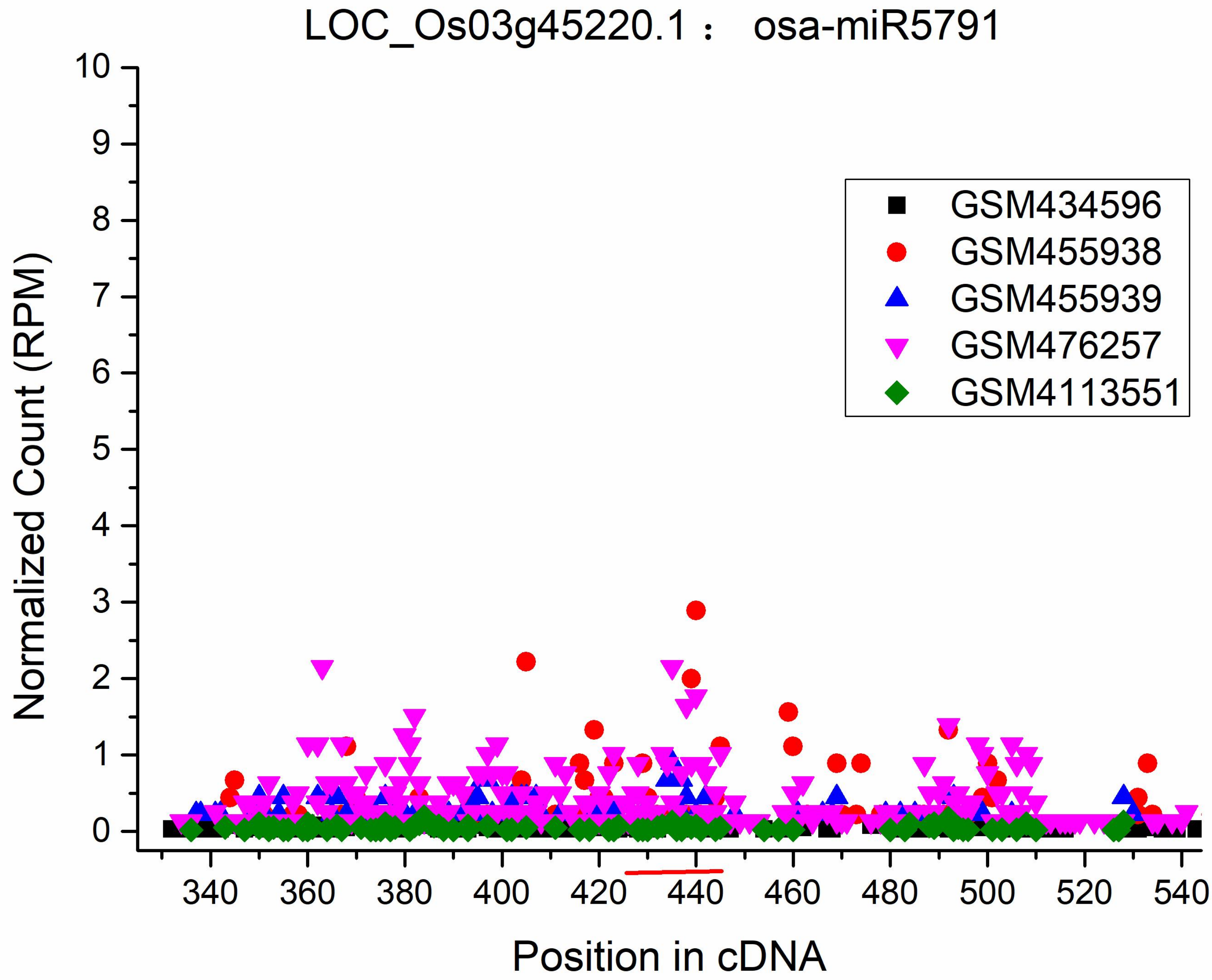
LOC_Os03g45220.1： osa-miR5791
Normalized Count (RPM)
Position in cDNA
0
1
2
3
4
5
6
7
8
9
10
340
360
380
400
420
440
460
480
500
520
540
GSM434596
GSM455938
GSM455939
GSM476257
GSM4113551

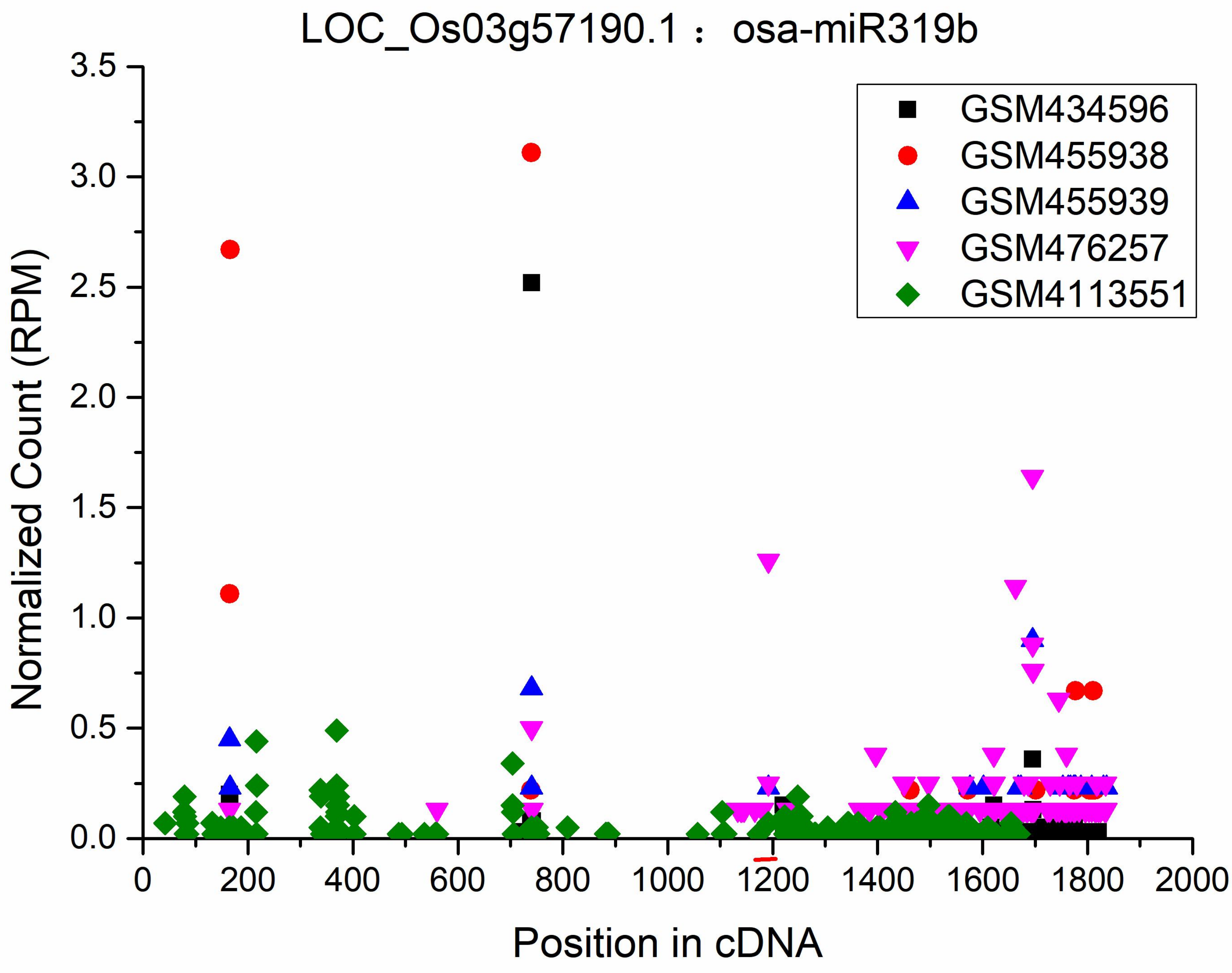
LOC_Os03g57190.1 ： osa-miR319b
Normalized Count (RPM)
Position in cDNA
GSM434596
GSM455938
GSM455939
GSM476257
GSM4113551
0.0
0.5
1.0
1.5
2.0
2.5
3.0
3.5
0
200
400
600
800
1000
1200
1400
1600
1800
2000

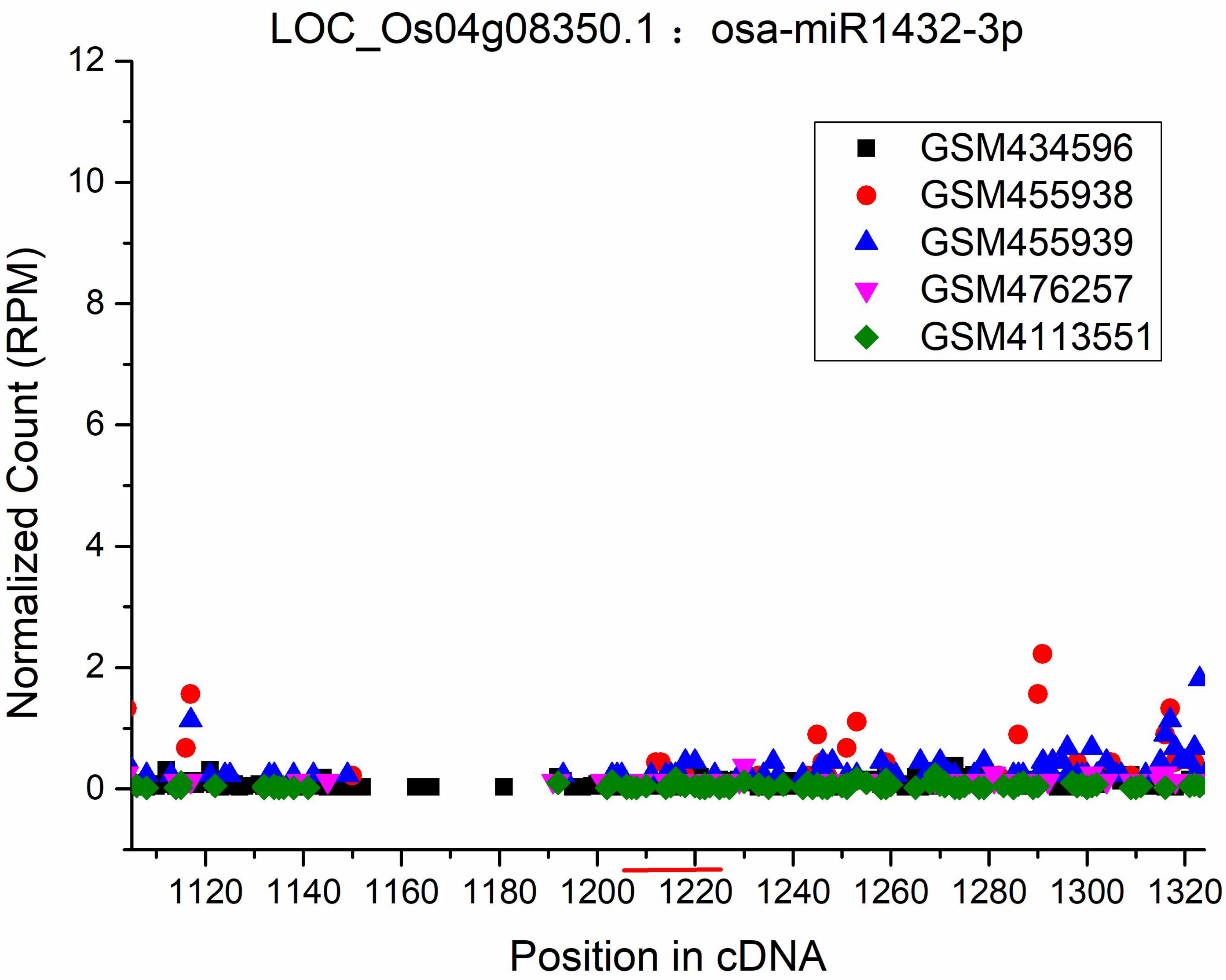
LOC_Os04g08350.1 ： osa-miR1432-3p
Normalized Count (RPM)
12
10
8
6
4
2
0
GSM434596
GSM455938
GSM455939
GSM476257
GSM4113551
1120
1140
1160
1180
1200
1220
1240
1260
1280
1300
1320
Position in cDNA

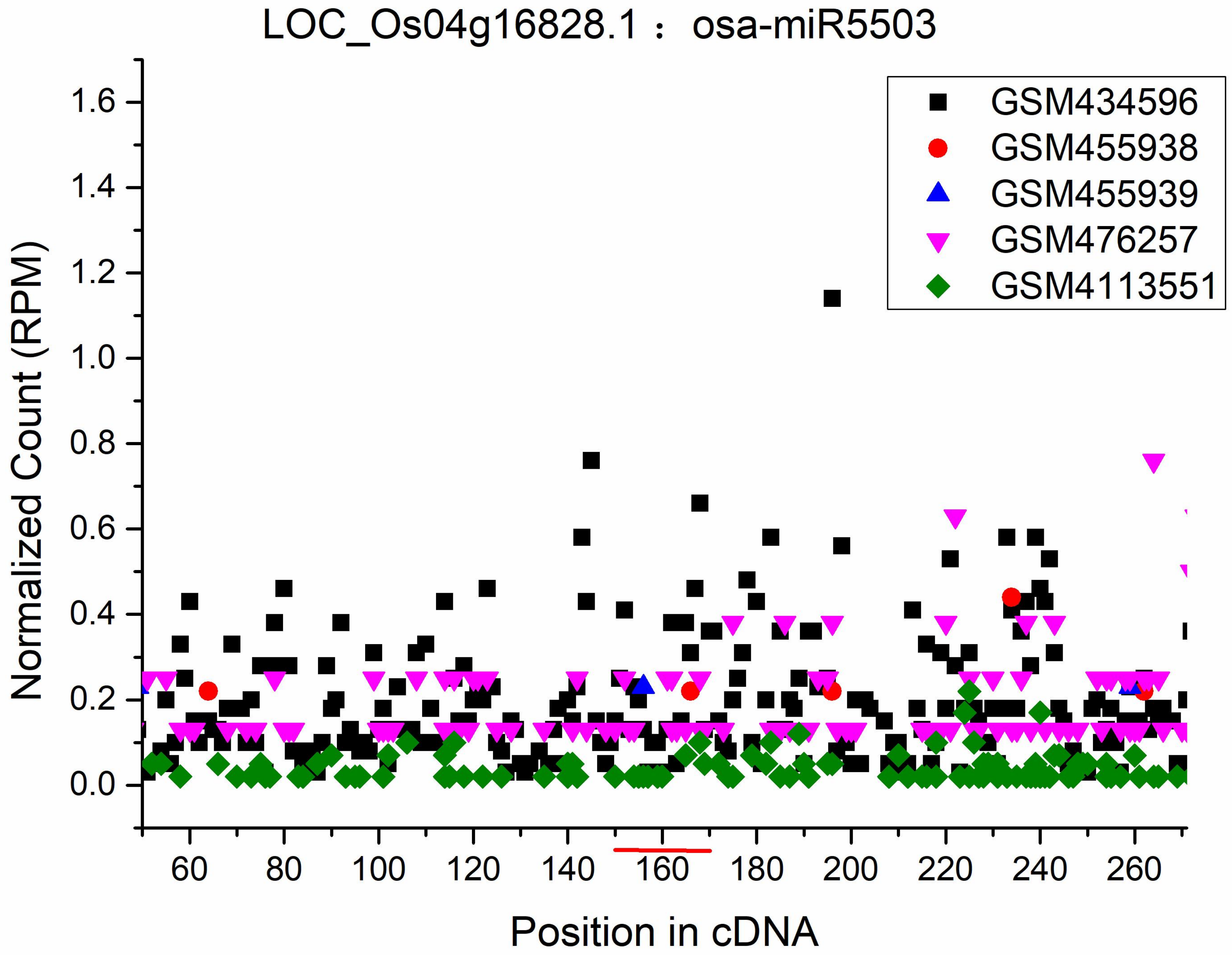
LOC_Os04g16828.1 ： osa-miR5503
Normalized Count (RPM)
Position in cDNA
GSM434596
GSM455938
GSM455939
GSM476257
GSM4113551
0.0
0.2
0.4
0.6
0.8
1.0
1.2
1.4
1.6
60
80
100
120
140
160
180
200
220
240
260

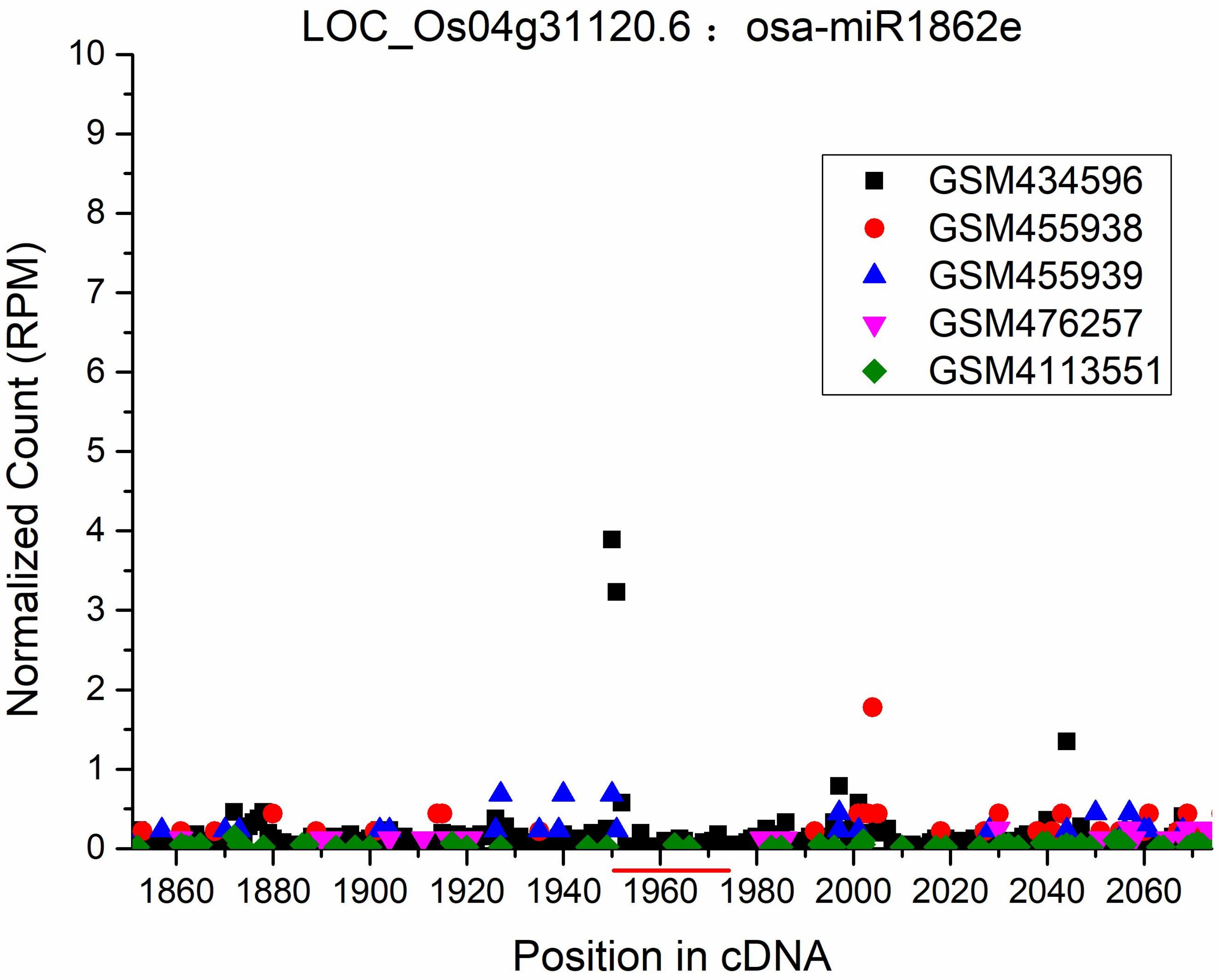

LOC_Os04g31120.6： osa-miR1862e
Normalized Count (RPM)
Position in cDNA
GSM434596
GSM455938
GSM455939
GSM476257
GSM4113551
0
1
2
3
4
5
6
7
8
9
10
1860
1880
1900
1920
1940
1960
1980
2000
2020
2040
2060

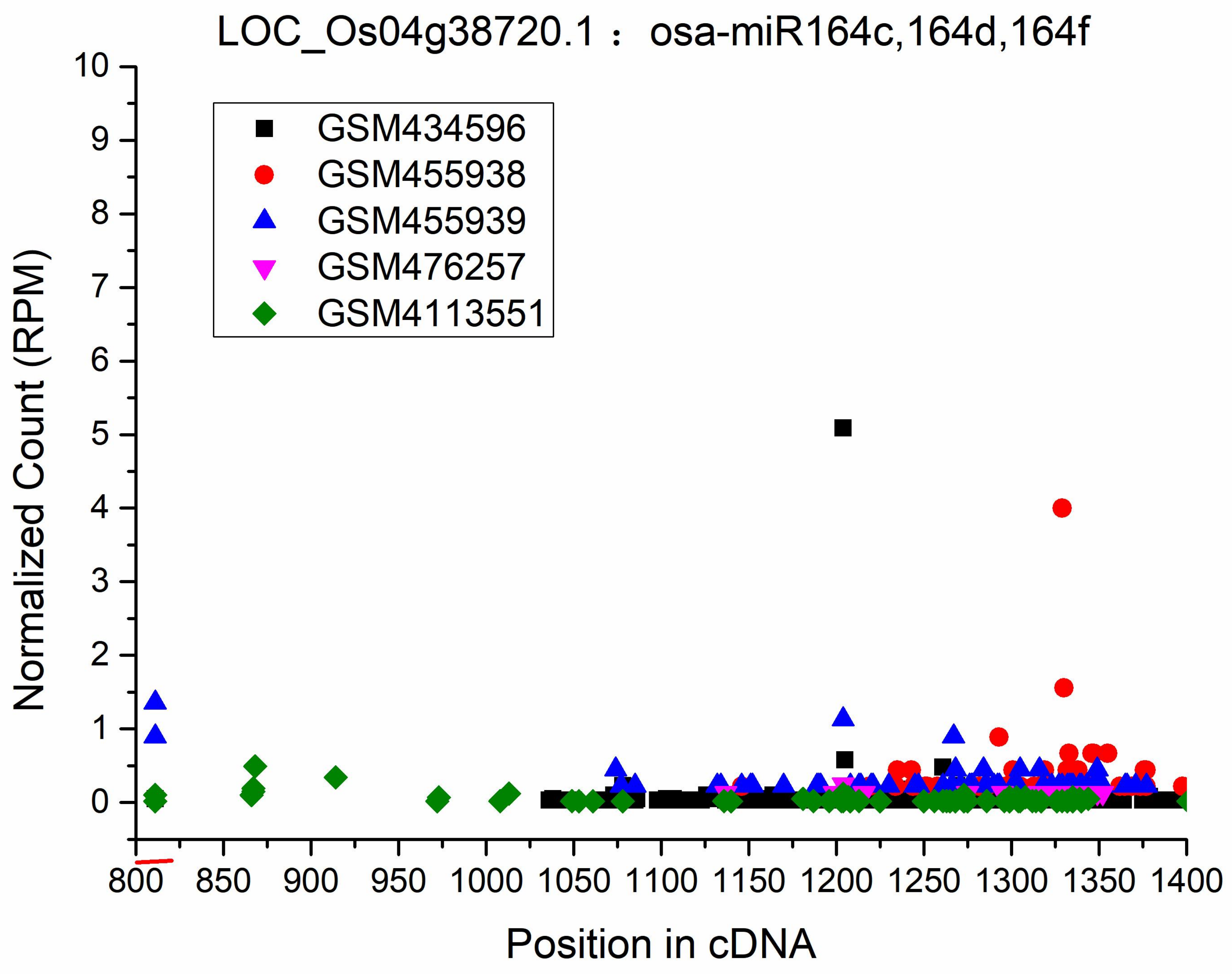
LOC_Os04g38720.1 ： osa-miR164c,164d,164f
GSM434596
GSM455938
GSM455939
GSM476257
GSM4113551
Normalized Count (RPM)
Position in cDNA
0
1
2
3
4
5
6
7
8
9
10
800
850
900
950
1000
1050
1100
1150
1200
1250
1300
1350
1400

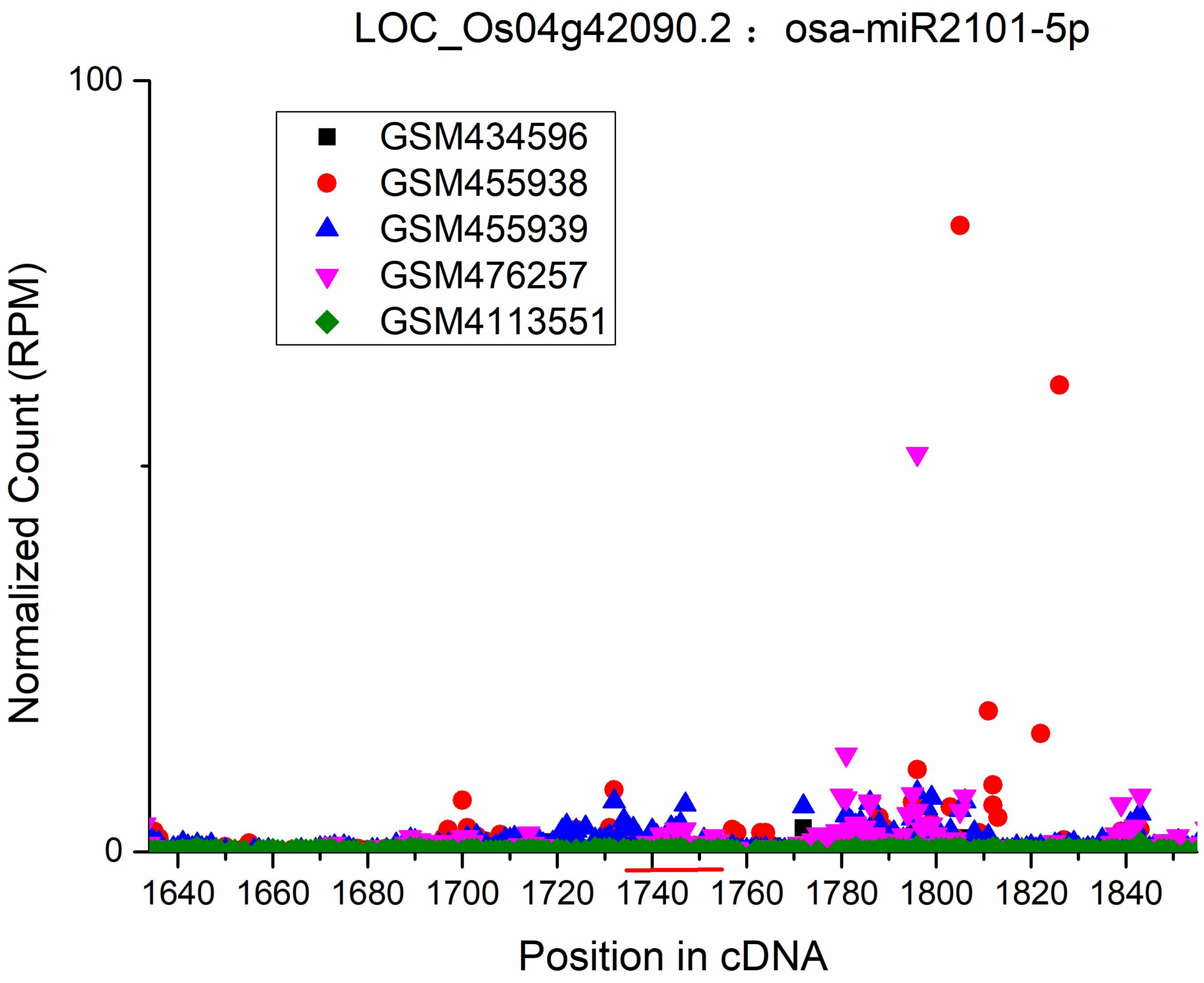

LOC_Os04g42090.2 ： osa-miR2101-5p
GSM434596
GSM455938
GSM455939
GSM476257
GSM4113551
100
0
Normalized Count (RPM)
1640
1660
1680
1700
1720
1740
1760
1780
1800
1820
1840
Position in cDNA

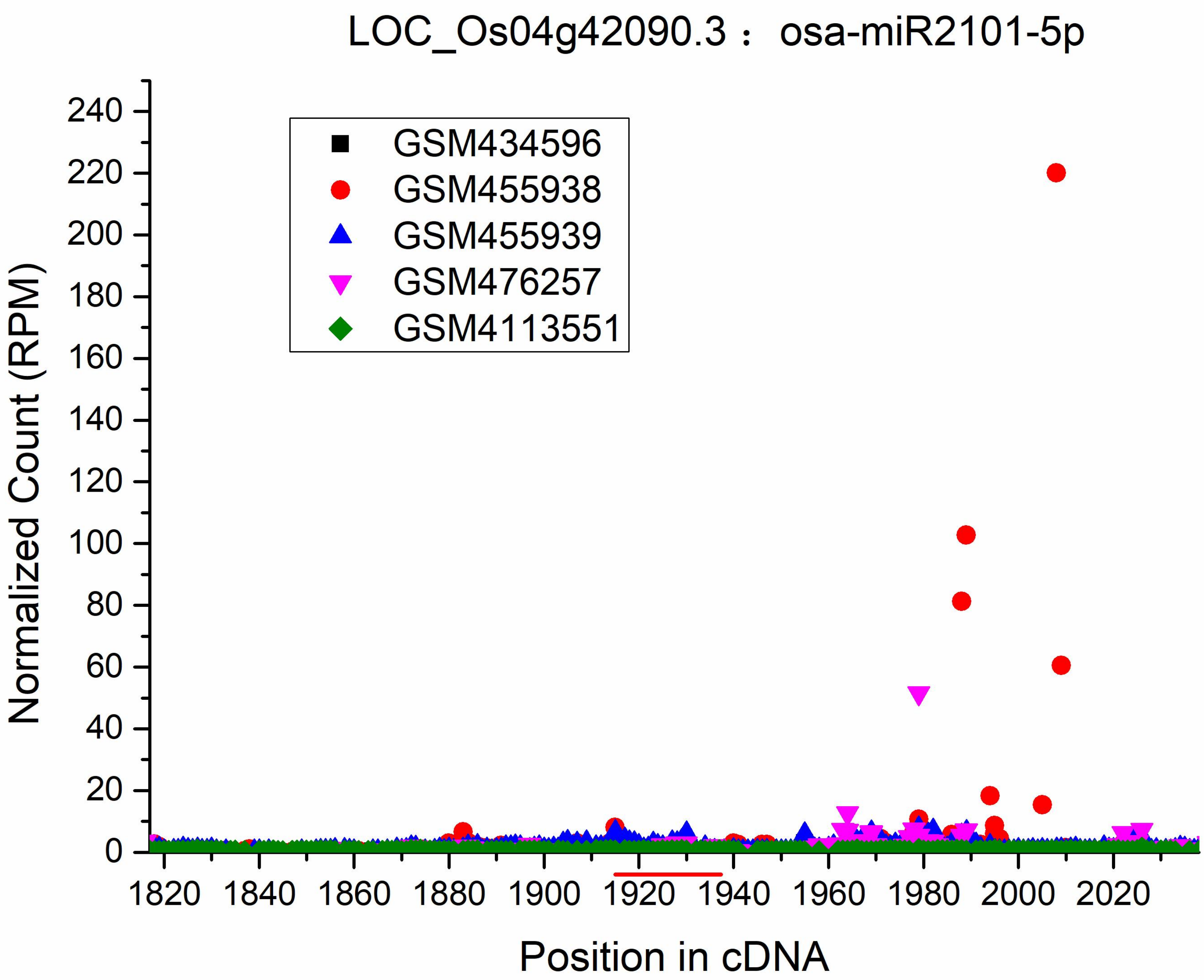

LOC_Os04g42090.3 : osa-miR2101-5p
Normalized Count (RPM)
Position in cDNA
GSM434596
GSM455938
GSM455939
GSM476257
GSM4113551
0
20
40
60
80
100
120
140
160
180
200
220
240
1820
1840
1860
1880
1900
1920
1940
1960
1980
2000
2020

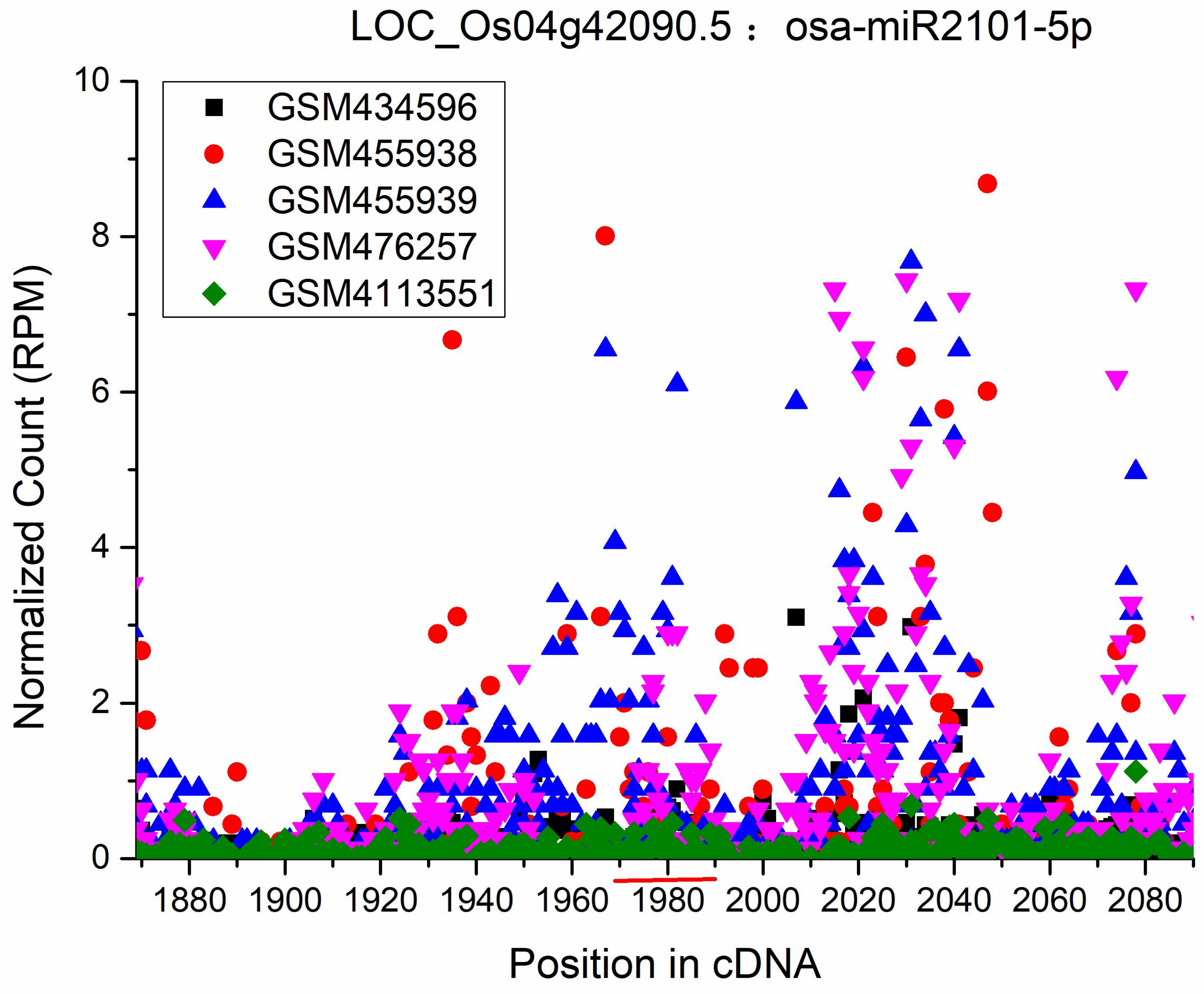

LOC_Os04g42090.5： osa-miR2101-5p
Normalized Count (RPM)
Position in cDNA
GSM434596
GSM455938
GSM455939
GSM476257
GSM4113551
0
2
4
6
8
10
1880
1900
1920
1940
1960
1980
2000
2020
2040
2060
2080

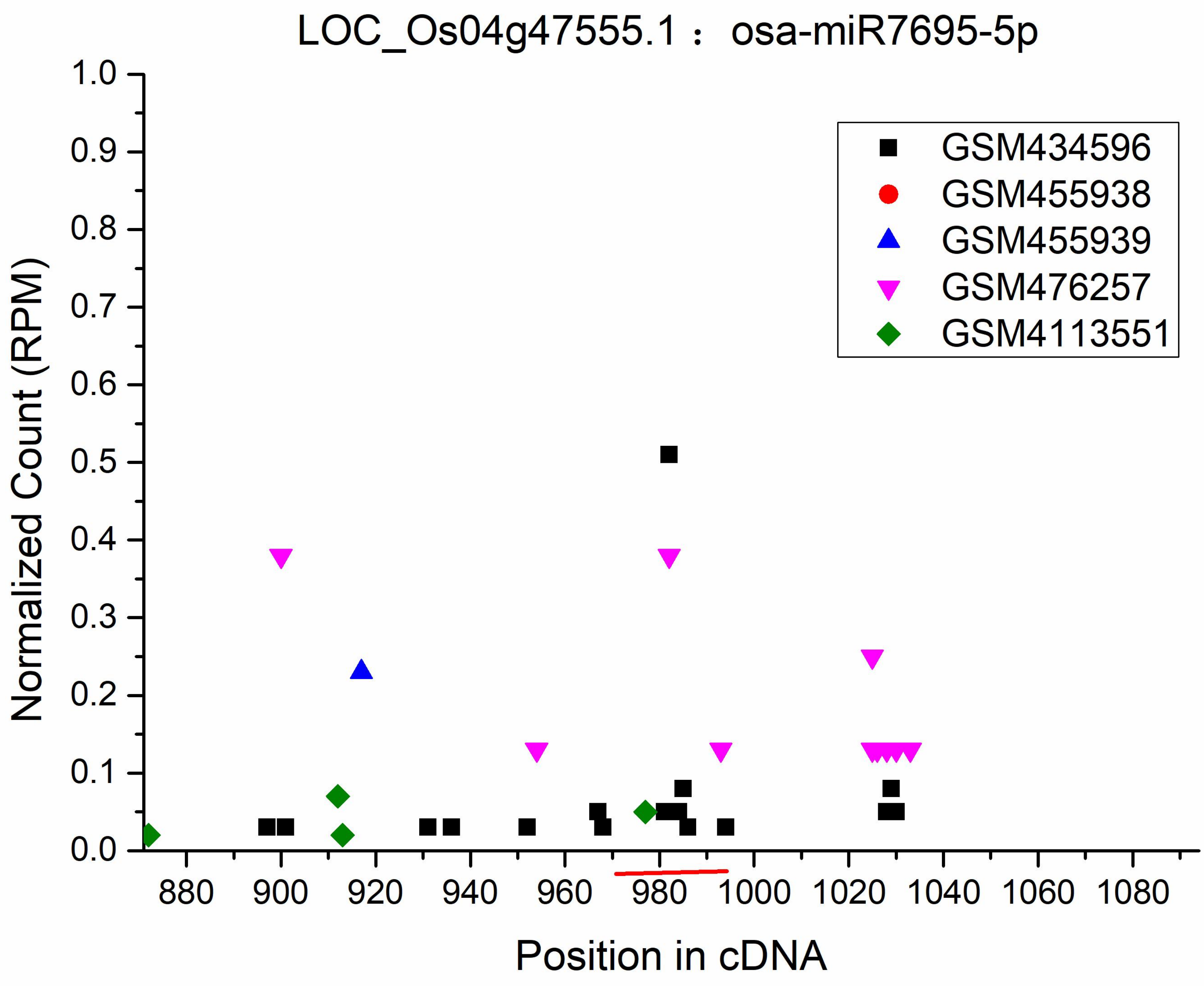
LOC_Os04g47555.1： osa-miR7695-5p
Normalized Count (RPM)
Position in cDNA
GSM434596
GSM455938
GSM455939
GSM476257
GSM4113551
0.0
0.1
0.2
0.3
0.4
0.5
0.6
0.7
0.8
0.9
1.0
880
900
920
940
960
980
1000
1020
1040
1060
1080

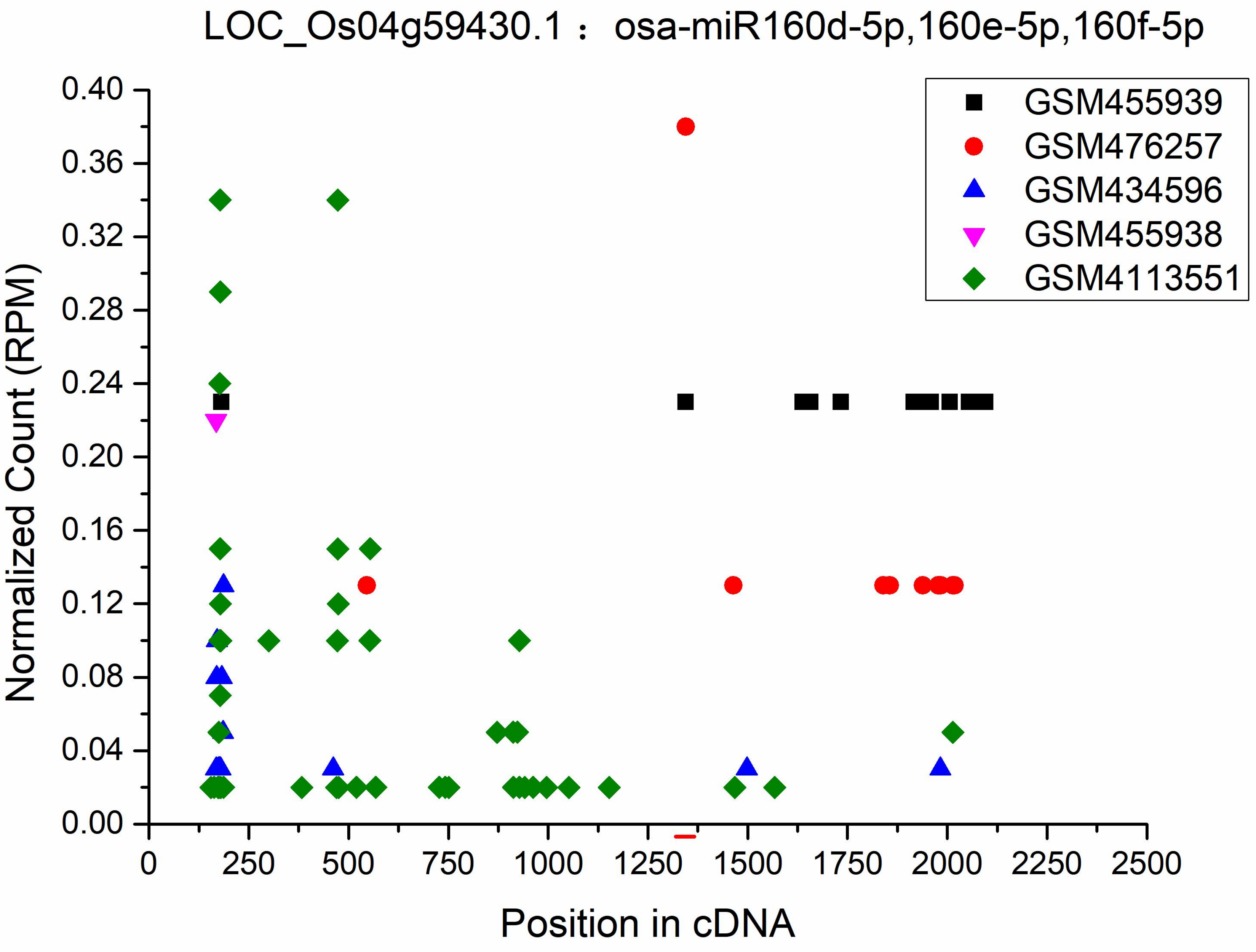
LOC_Os04g59430.1：osa-miR160d-5p,160e-5p,160f-5p
Normalized Count (RPM)
Position in cDNA
0.00
0.04
0.08
0.12
0.16
0.20
0.24
0.28
0.32
0.36
0.40
0
250
500
750
1000
1250
1500
1750
2000
2250
2500
GSM455939
GSM476257
GSM434596
GSM455938
GSM4113551

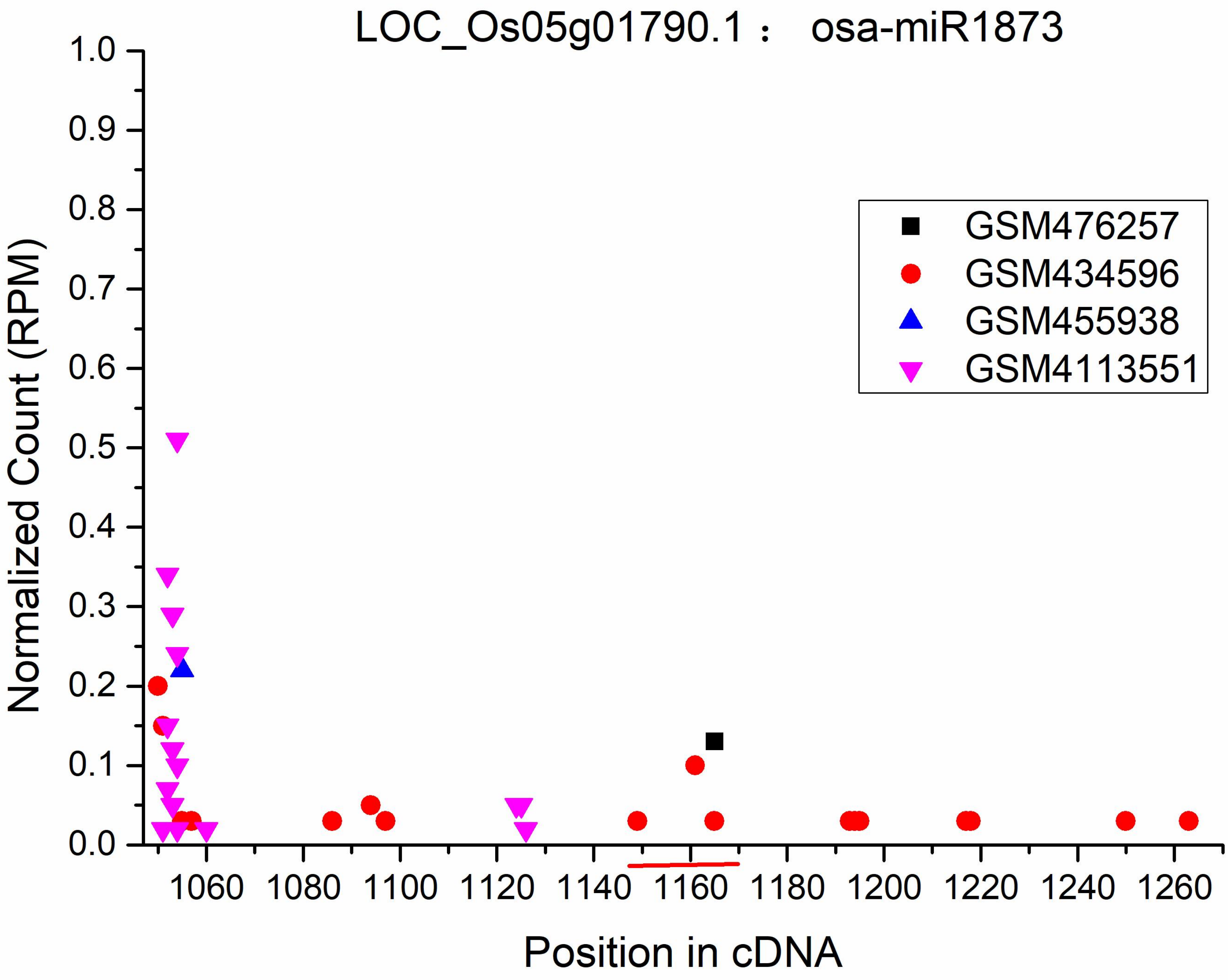
LOC_Os05g01790.1： osa-miR1873
Normalized Count (RPM)
1.0
0.9
0.8
0.7
0.6
0.5
0.4
0.3
0.2
0.1
0.0
1060
1080
1100
1120
1140
1160
1180
1200
1220
1240
1260
Position in cDNA
GSM476257
GSM434596
GSM455938
GSM4113551

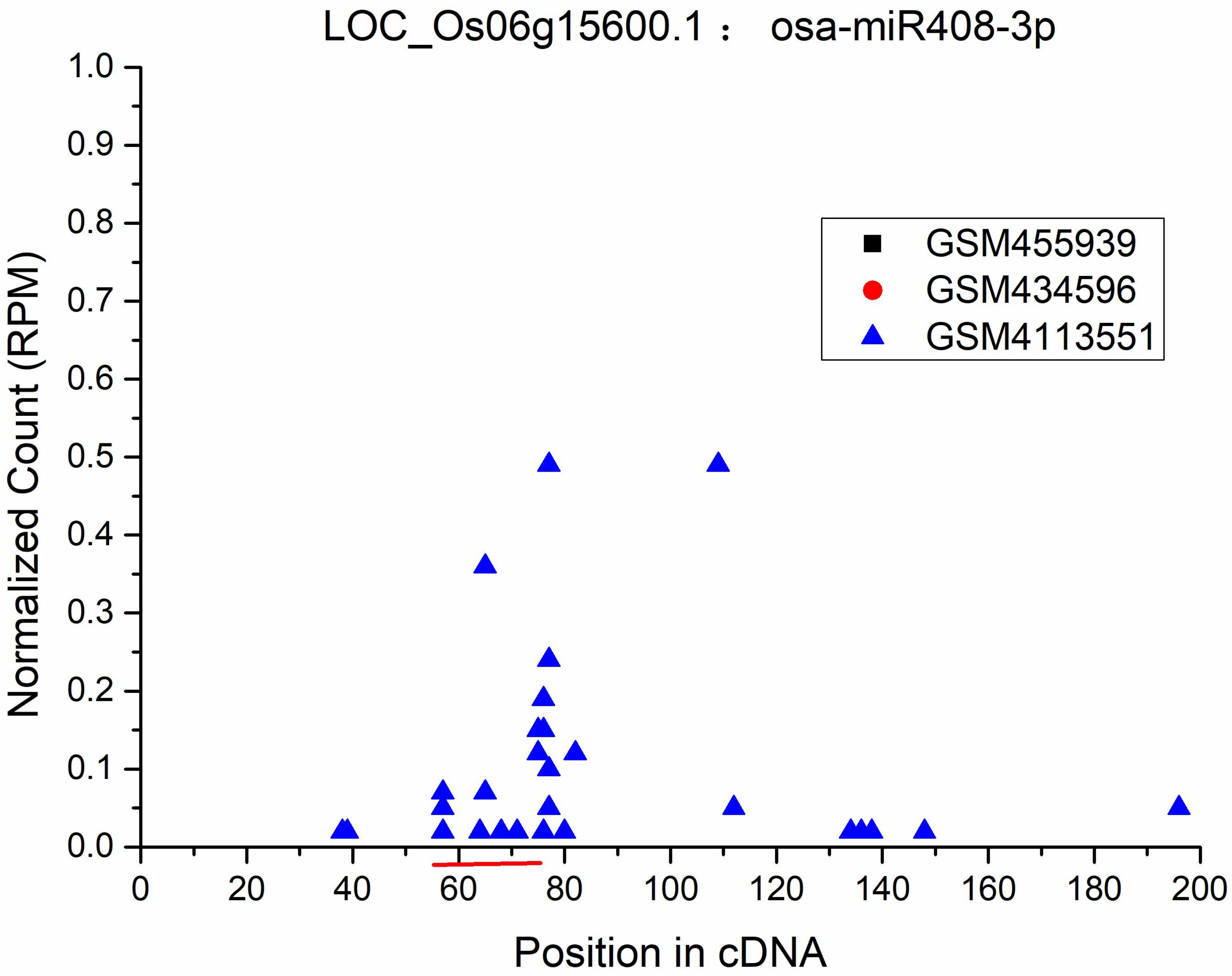

LOC_Os06g15600.1 ： osa-miR408-3p
Normalized Count (RPM)
1.0
0.9
0.8
0.7
0.6
0.5
0.4
0.3
0.2
0.1
0.0
0
20
40
60
80
100
120
140
160
180
200
Position in cDNA
GSM455939
GSM434596
GSM4113551

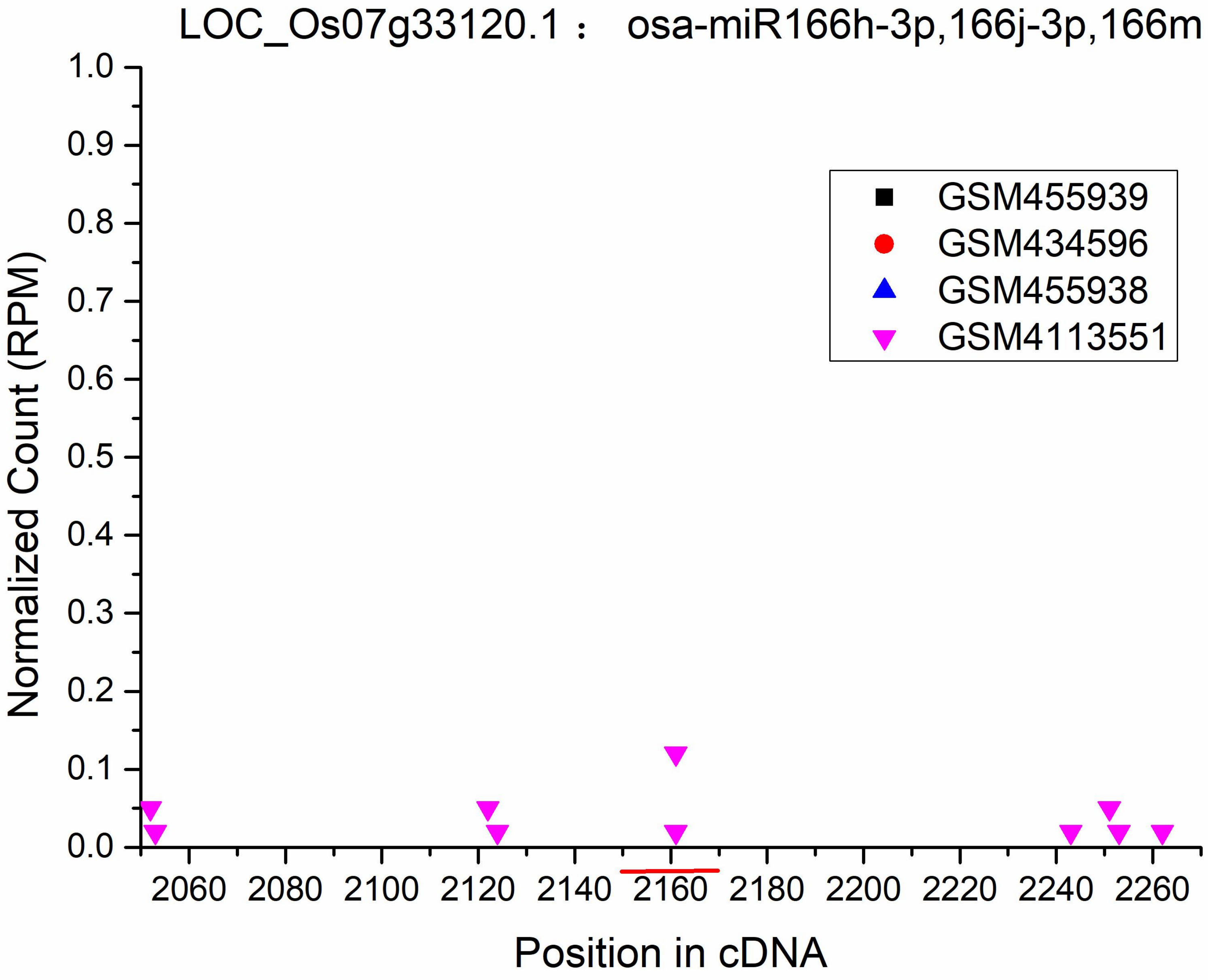
LOC_Os07g33120.1： osa-miR166h-3p,166j-3p,166m
Normalized Count (RPM)
1.0
0.9
0.8
0.7
0.6
0.5
0.4
0.3
0.2
0.1
0.0
GSM455939
GSM434596
GSM455938
GSM4113551
2060
2080
2100
2120
2140
2160
2180
2200
2220
2240
2260
Position in cDNA

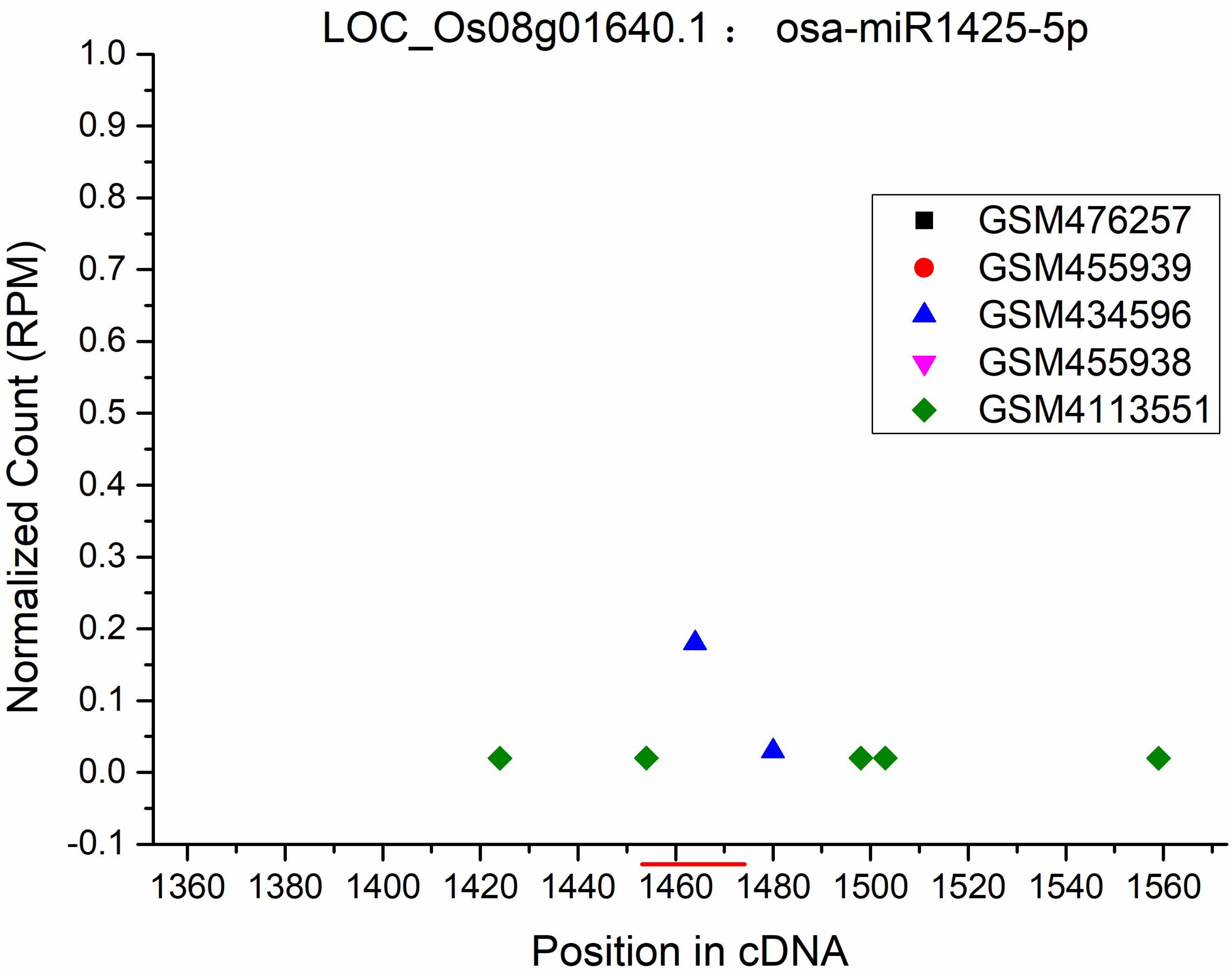

LOC_Os08g01640.1 ： osa-miR1425-5p
Normalized Count (RPM)
1.0
0.9
0.8
0.7
0.6
0.5
0.4
0.3
0.2
0.1
0.0
-0.1
1360
1380
1400
1420
1440
1460
1480
1500
1520
1540
1560
Position in cDNA
GSM476257
GSM455939
GSM434596
GSM455938
GSM4113551

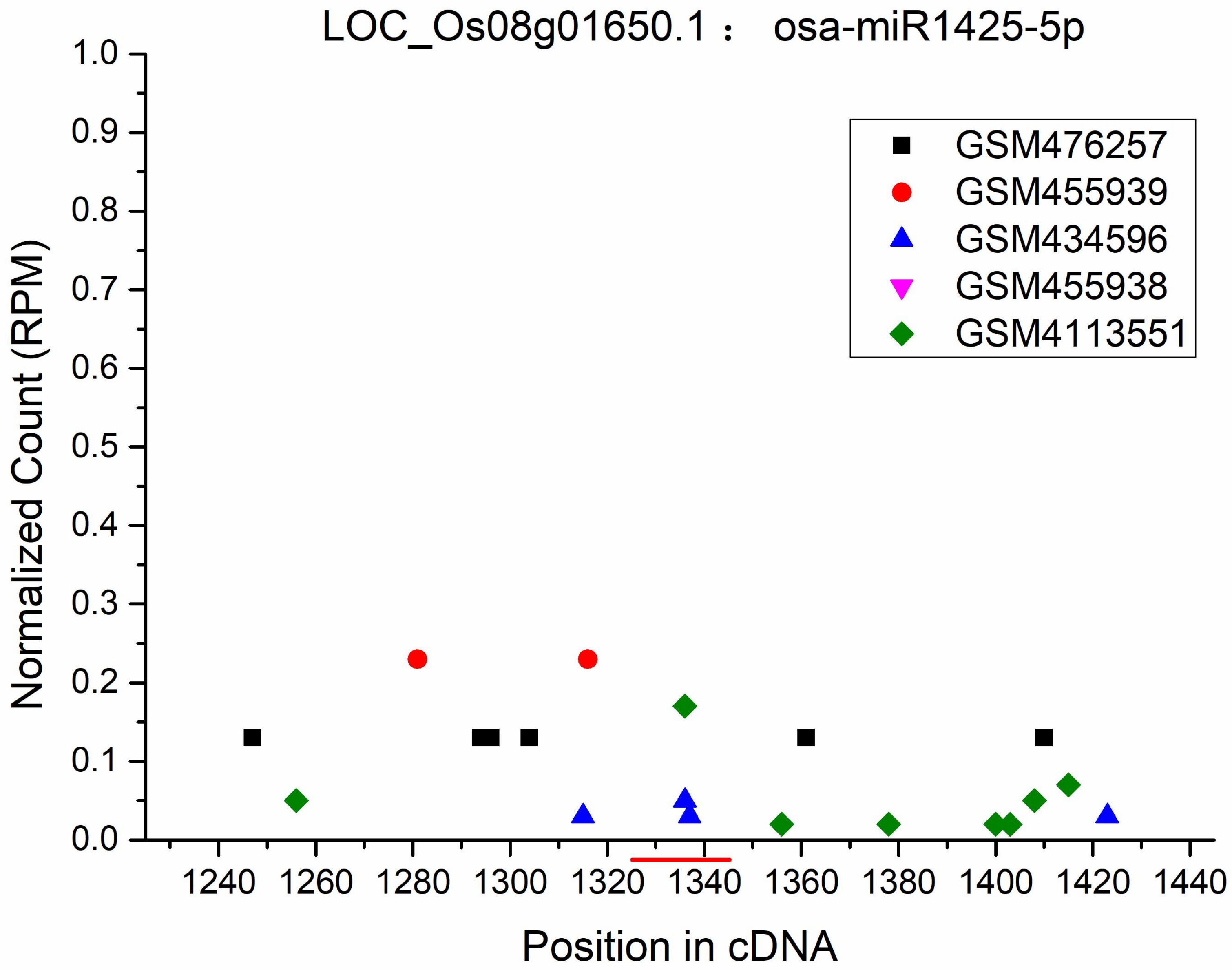
LOC_Os08g01650.1： osa-miR1425-5p
Normalized Count (RPM)
1.0
0.9
0.8
0.7
0.6
0.5
0.4
0.3
0.2
0.1
0.0
1240
1260
1280
1300
1320
1340
1360
1380
1400
1420
1440
Position in cDNA
GSM476257
GSM455939
GSM434596
GSM455938
GSM4113551

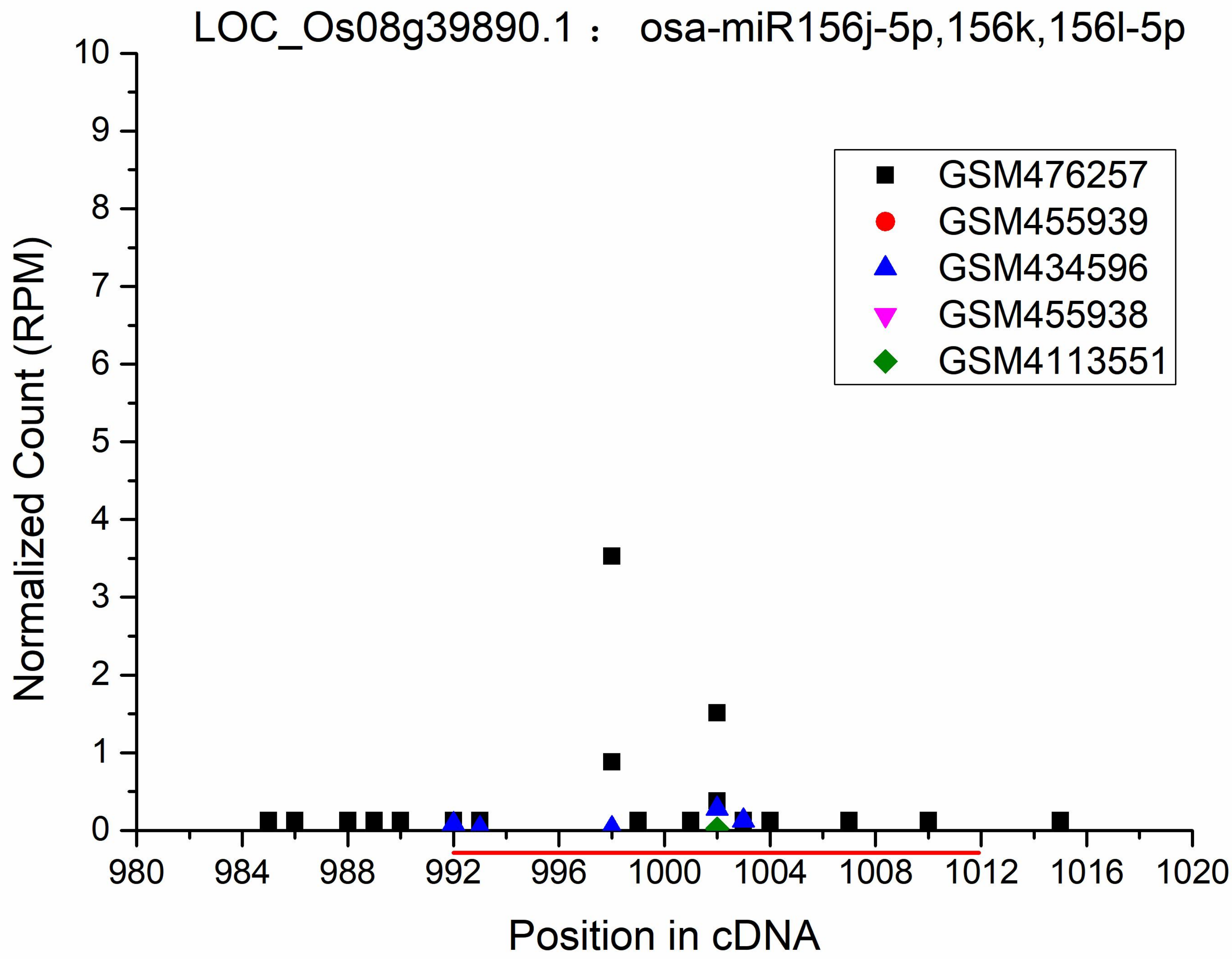

LOC_Os08g39890.1 ： osa-miR156j-5p,156k,156l-5p
Normalized Count (RPM)
0
1
2
3
4
5
6
7
8
9
10
980
984
988
992
996
1000
1004
1008
1012
1016
1020
Position in cDNA
GSM476257
GSM455939
GSM434596
GSM455938
GSM4113551

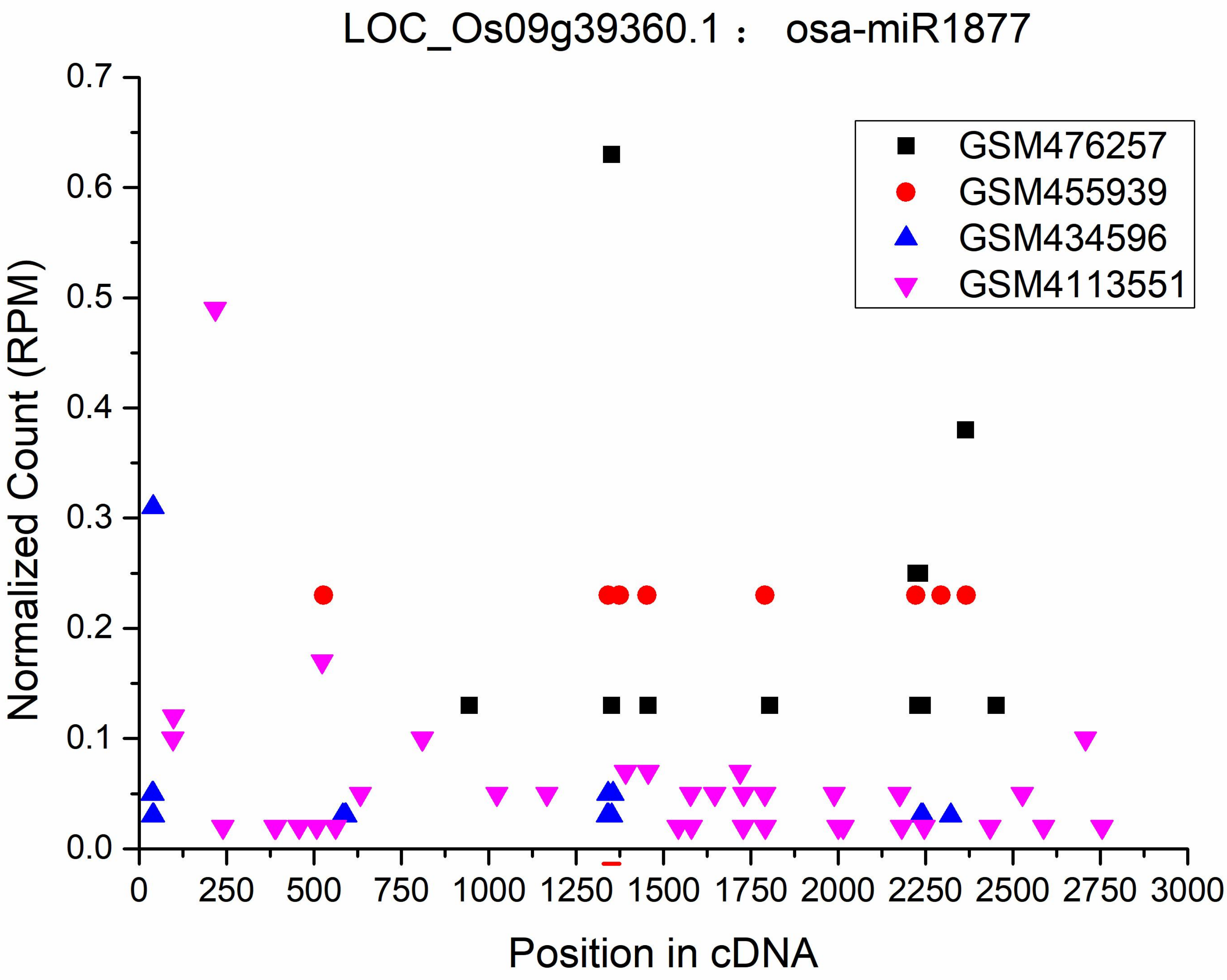
LOC_Os09g39360.1： osa-miR1877
Normalized Count (RPM)
0.0
0.1
0.2
0.3
0.4
0.5
0.6
0.7
0
250
500
750
1000
1250
1500
1750
2000
2250
2500
2750
3000
Position in cDNA
GSM476257
GSM455939
GSM434596
GSM4113551

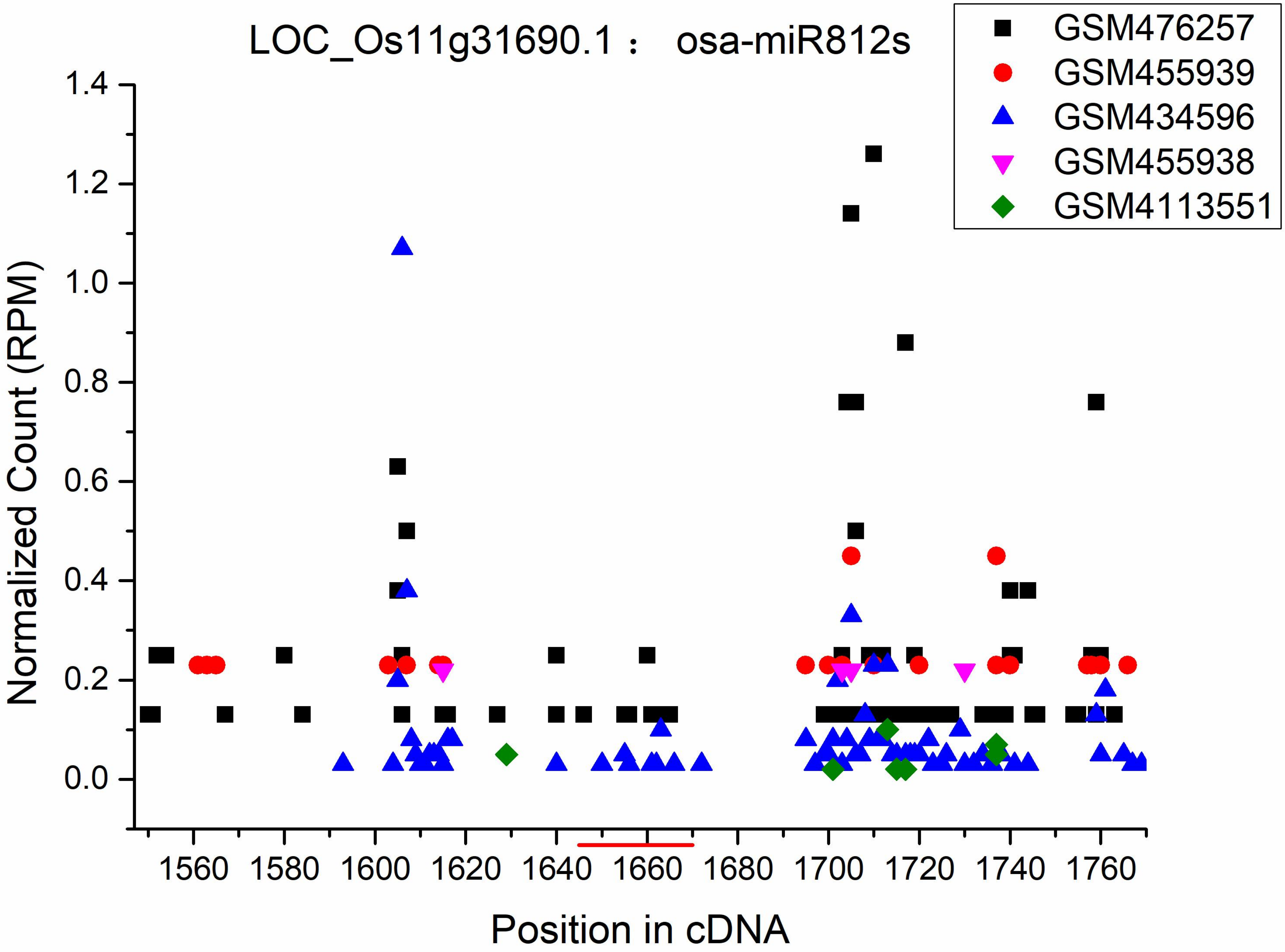

LOC_Os11g31690.1 ： osa-miR812s
GSM476257
GSM455939
GSM434596
GSM455938
GSM4113551
Normalized Count (RPM)
0.0
0.2
0.4
0.6
0.8
1.0
1.2
1.4
1560
1580
1600
1620
1640
1660
1680
1700
1720
1740
1760
Position in cDNA

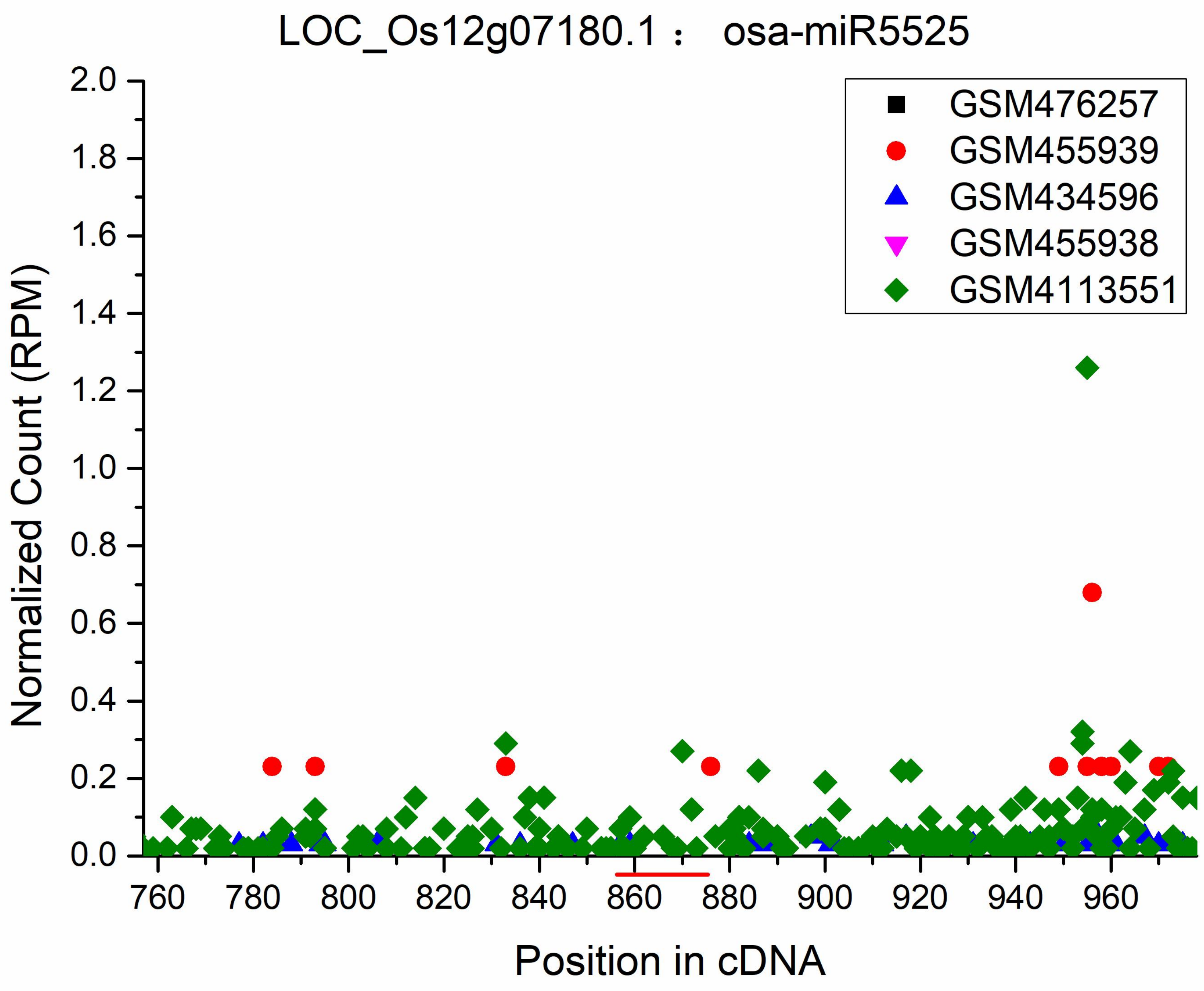

LOC_Os12g07180.1 ： osa-miR5525
GSM476257
GSM455939
GSM434596
GSM455938
GSM4113551
Normalized Count (RPM)
Position in cDNA
0.0
0.2
0.4
0.6
0.8
1.0
1.2
1.4
1.6
1.8
2.0
760
780
800
820
840
860
880
900
920
940
960

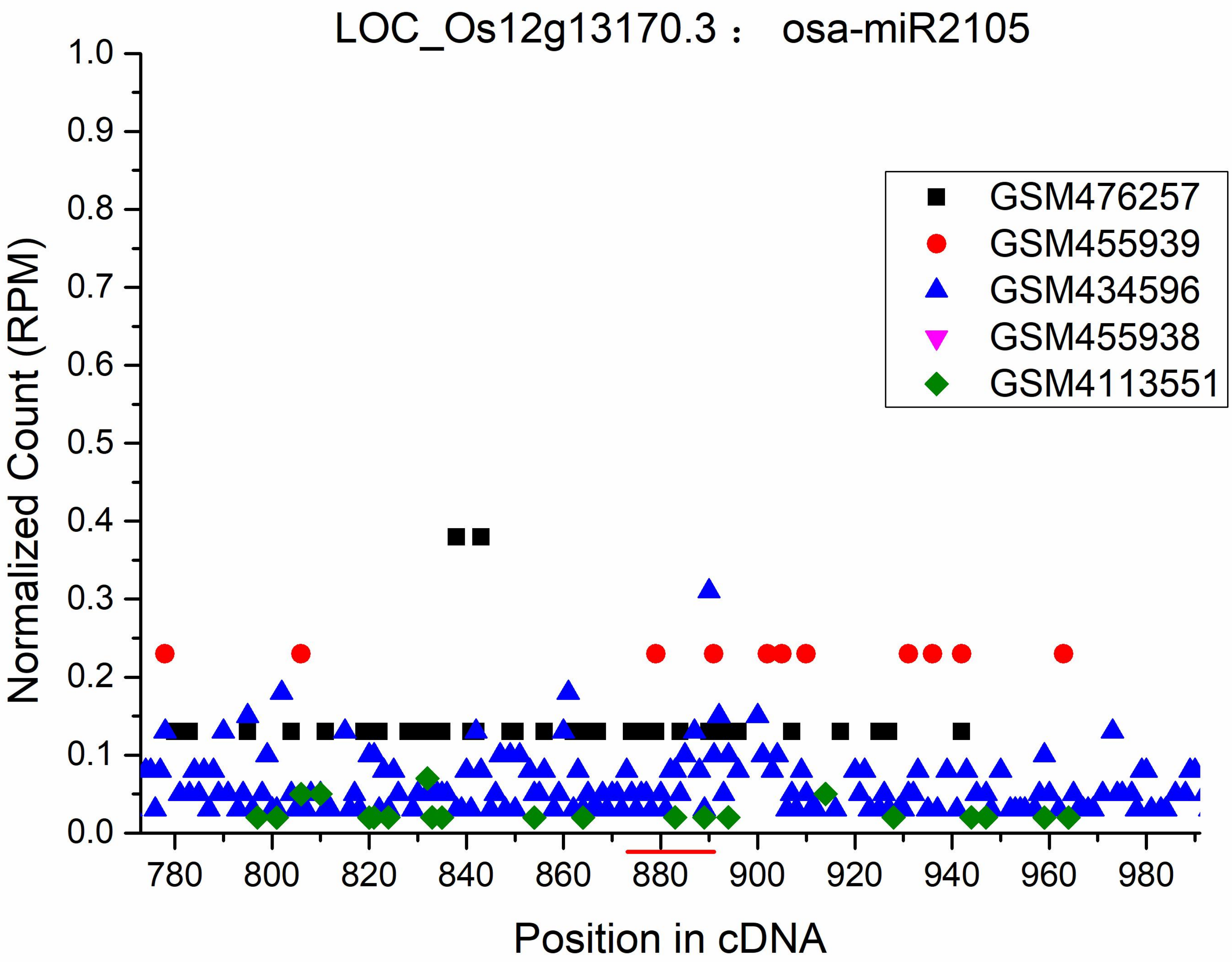

LOC_Os12g13170.3 ： osa-miR2105
Normalized Count (RPM)
1.0
0.9
0.8
0.7
0.6
0.5
0.4
0.3
0.2
0.1
0.0
780
800
820
840
860
880
900
920
940
960
980
Position in cDNA
GSM476257
GSM455939
GSM434596
GSM455938
GSM4113551

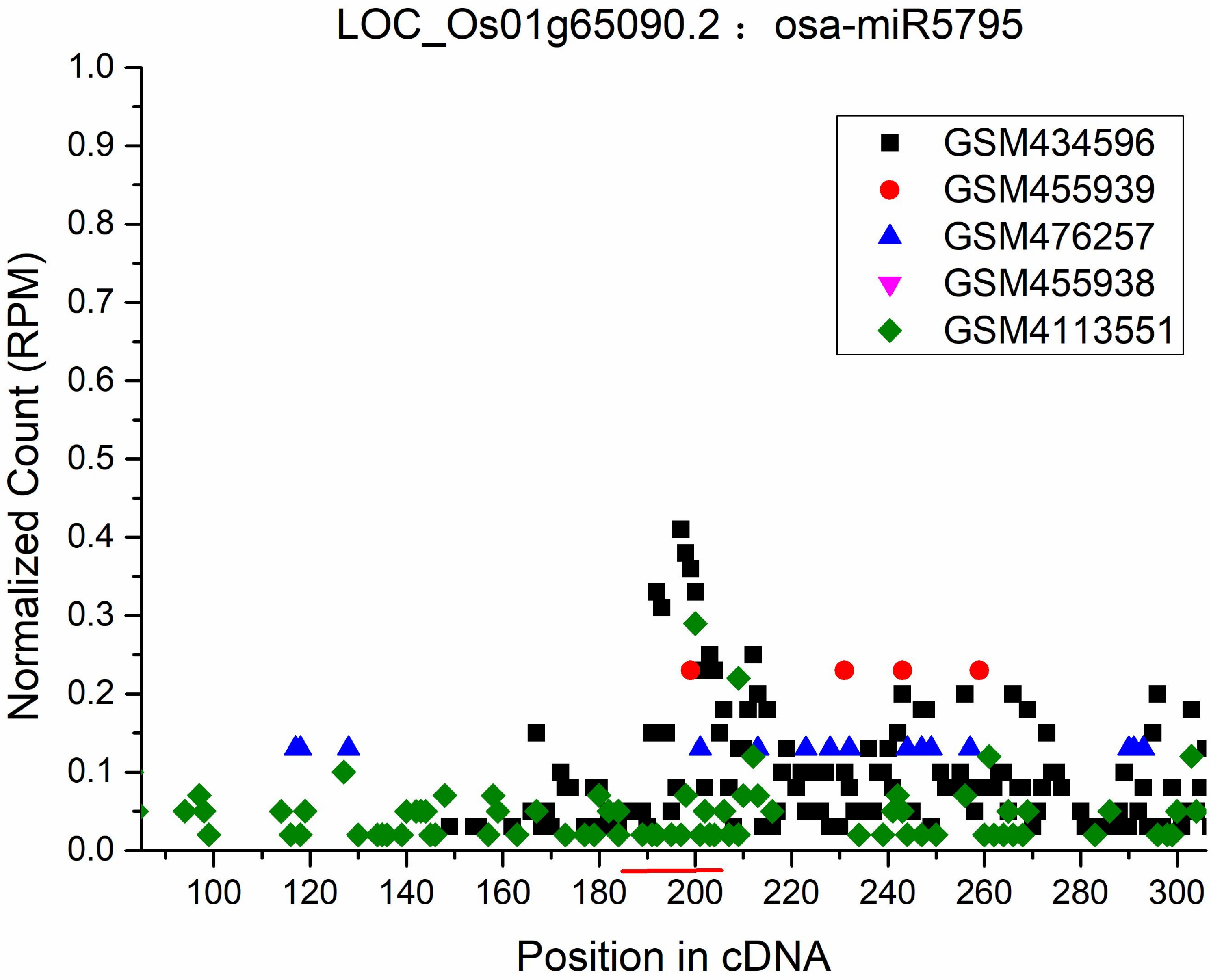

LOC_Os01g65090.2： osa-miR5795
Normalized Count (RPM)
1.0
0.9
0.8
0.7
0.6
0.5
0.4
0.3
0.2
0.1
0.0
100
120
140
160
180
200
220
240
260
280
300
Position in cDNA
GSM434596
GSM455939
GSM476257
GSM455938
GSM4113551

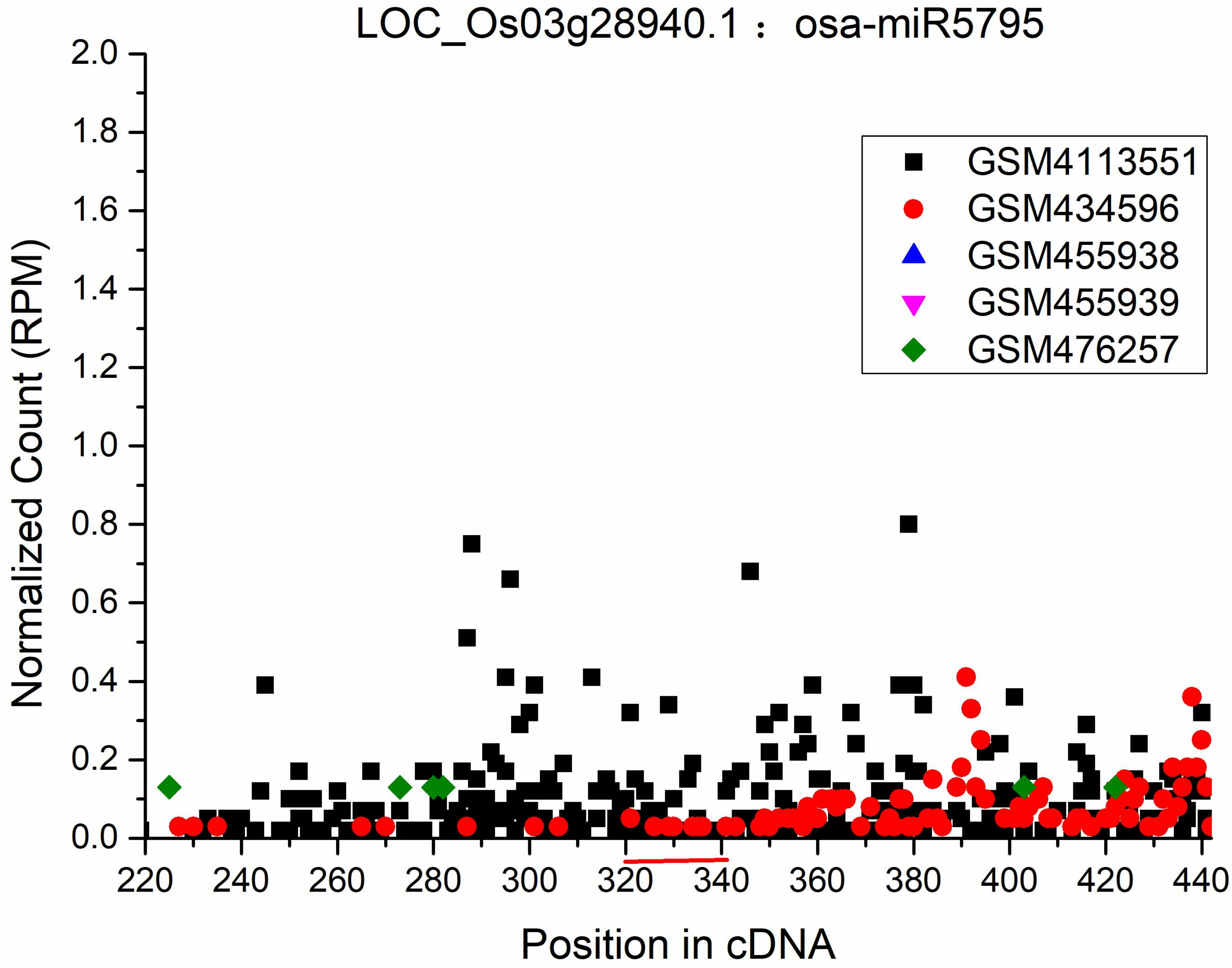

LOC_Os03g28940.1 ： osa-miR5795
Normalized Count (RPM)
2.0
1.8
1.6
1.4
1.2
1.0
0.8
0.6
0.4
0.2
0.0
220
240
260
280
300
320
340
360
380
400
420
440
Position in cDNA
GSM4113551
GSM434596
GSM455938
GSM455939
GSM476257

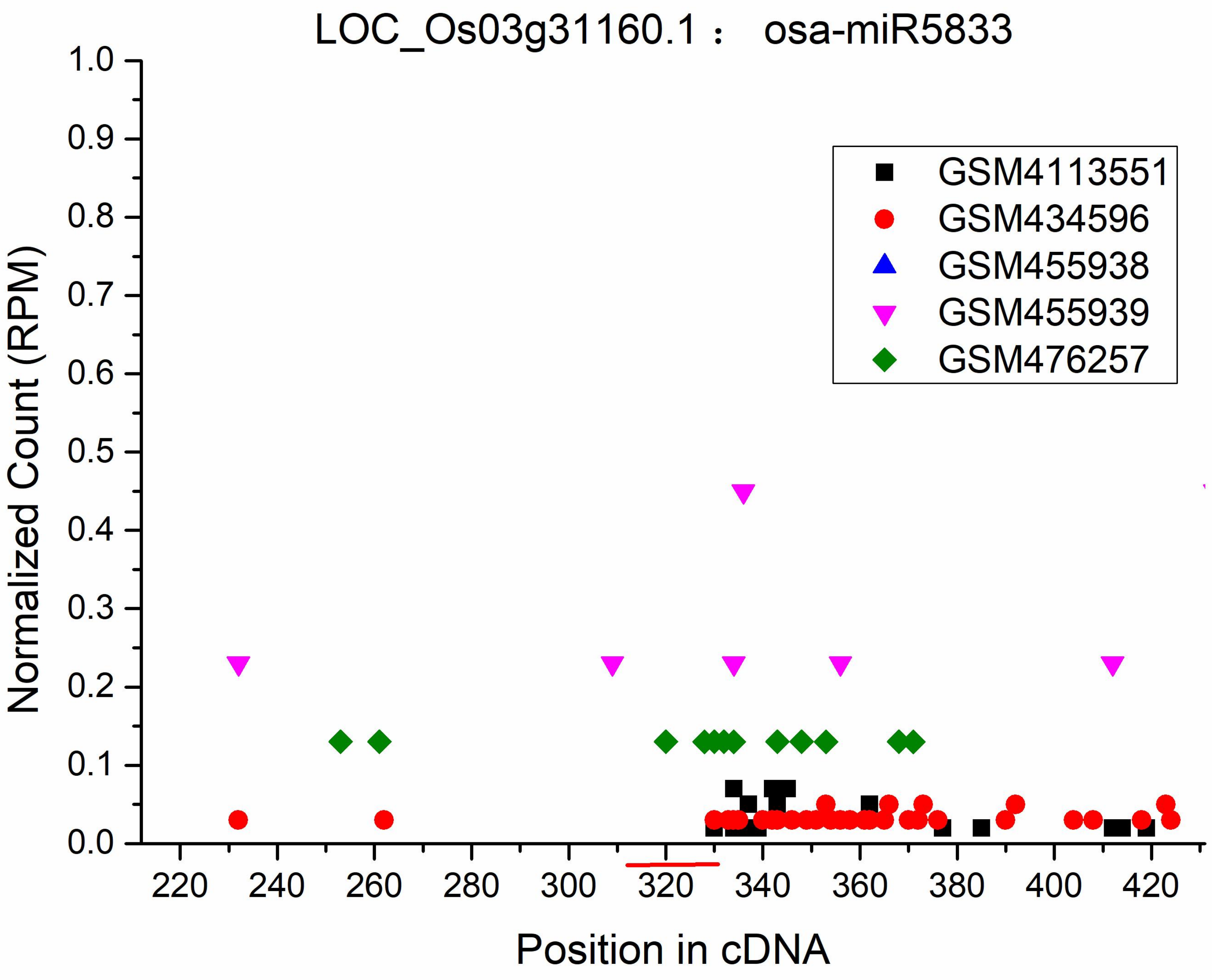
LOC_Os03g31160.1： osa-miR5833
Normalized Count (RPM)
1.0
0.9
0.8
0.7
0.6
0.5
0.4
0.3
0.2
0.1
0.0
220
240
260
280
300
320
340
360
380
400
420
Position in cDNA
GSM4113551
GSM434596
GSM455938
GSM455939
GSM476257

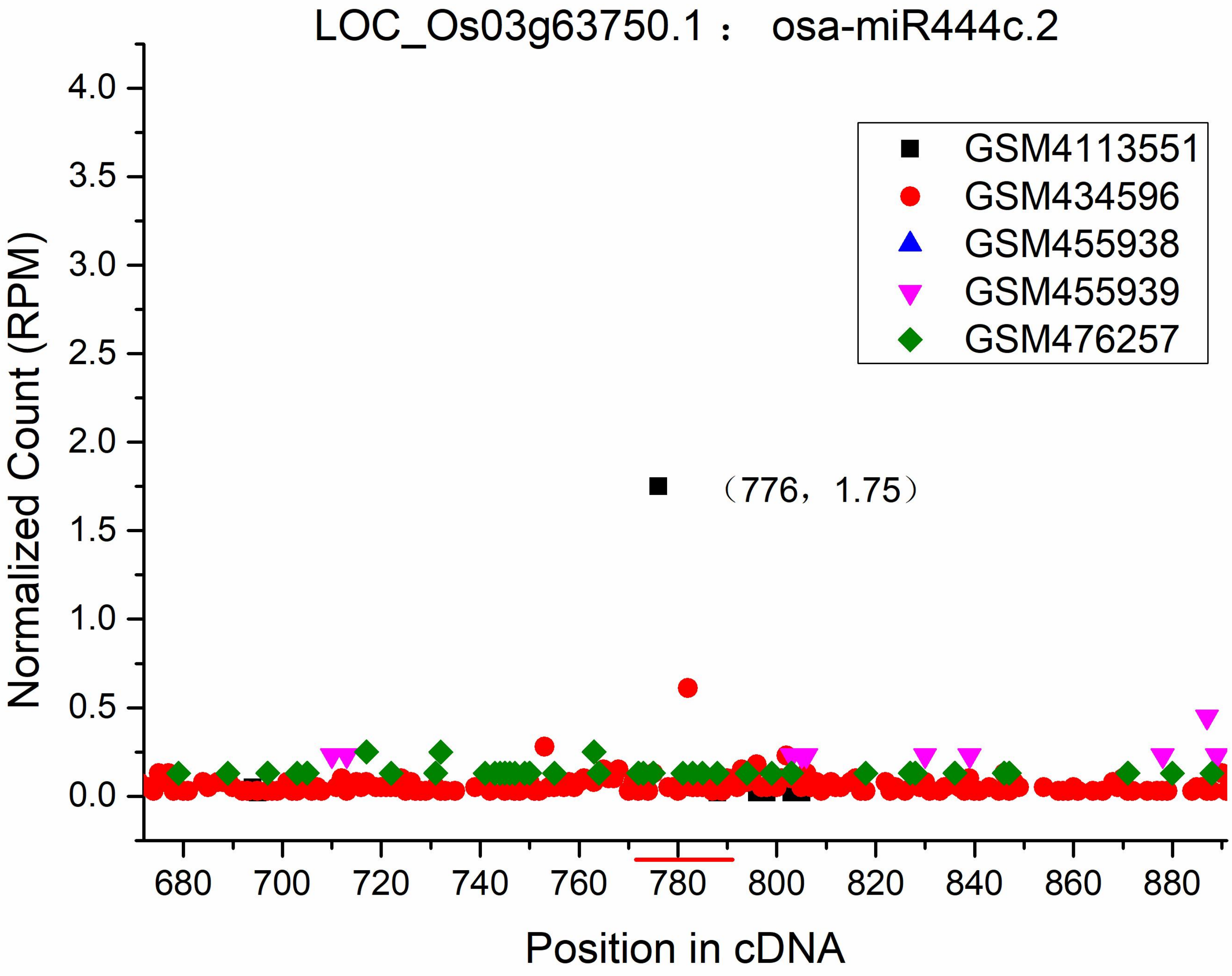
LOC_Os03g63750.1： osa-miR444c.2
Normalized Count (RPM)
4.0
3.5
3.0
2.5
2.0
1.5
1.0
0.5
0.0
GSM4113551
GSM434596
GSM455938
GSM455939
GSM476257
（776，1.75）
680
700
720
740
760
780
800
820
840
860
880
Position in cDNA

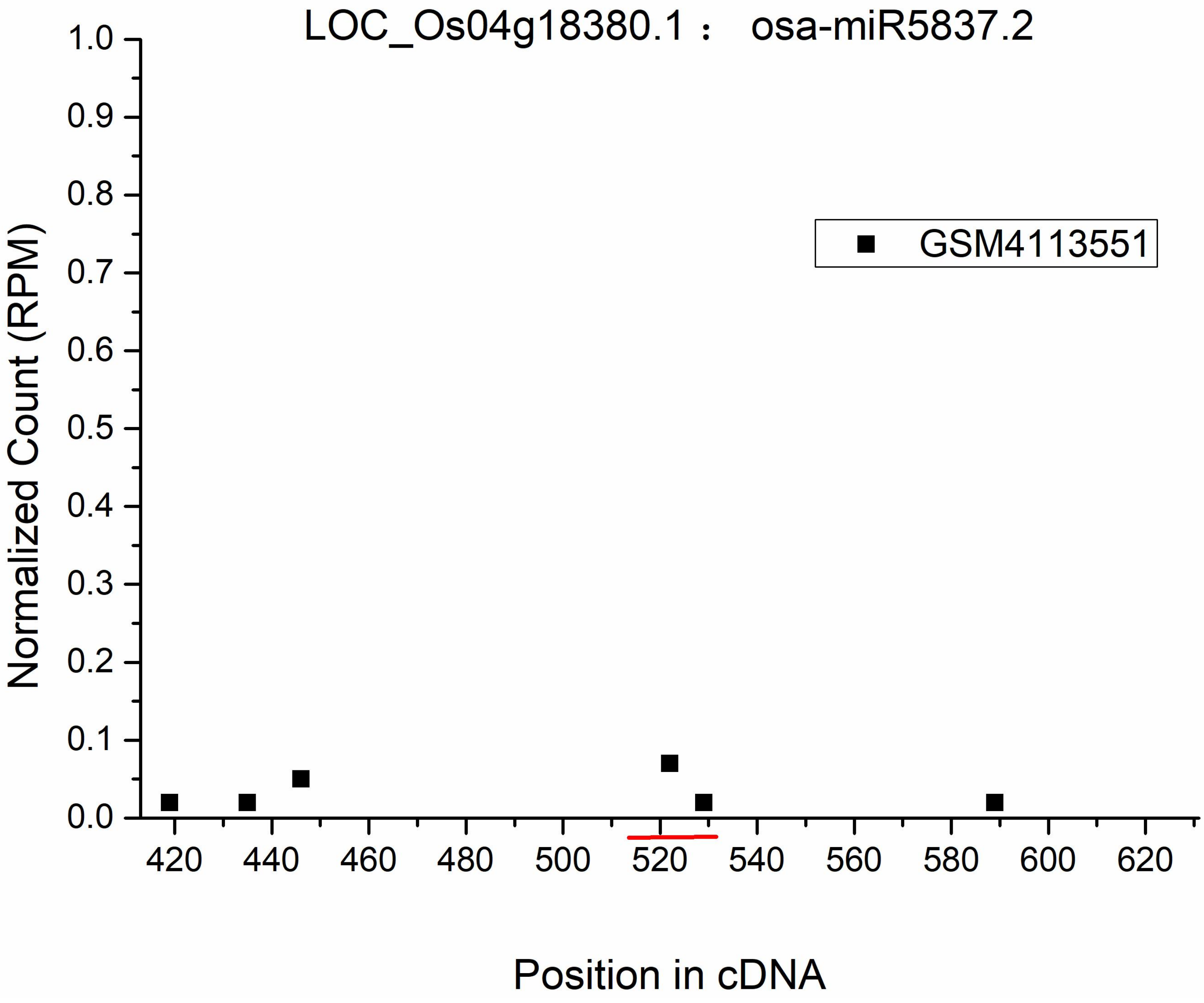
LOC_Os04g18380.1 ： osa-miR5837.2
Normalized Count (RPM)
1.0
0.9
0.8
0.7
0.6
0.5
0.4
0.3
0.2
0.1
0.0
GSM4113551
420
440
460
480
500
520
540
560
580
600
620
Position in cDNA

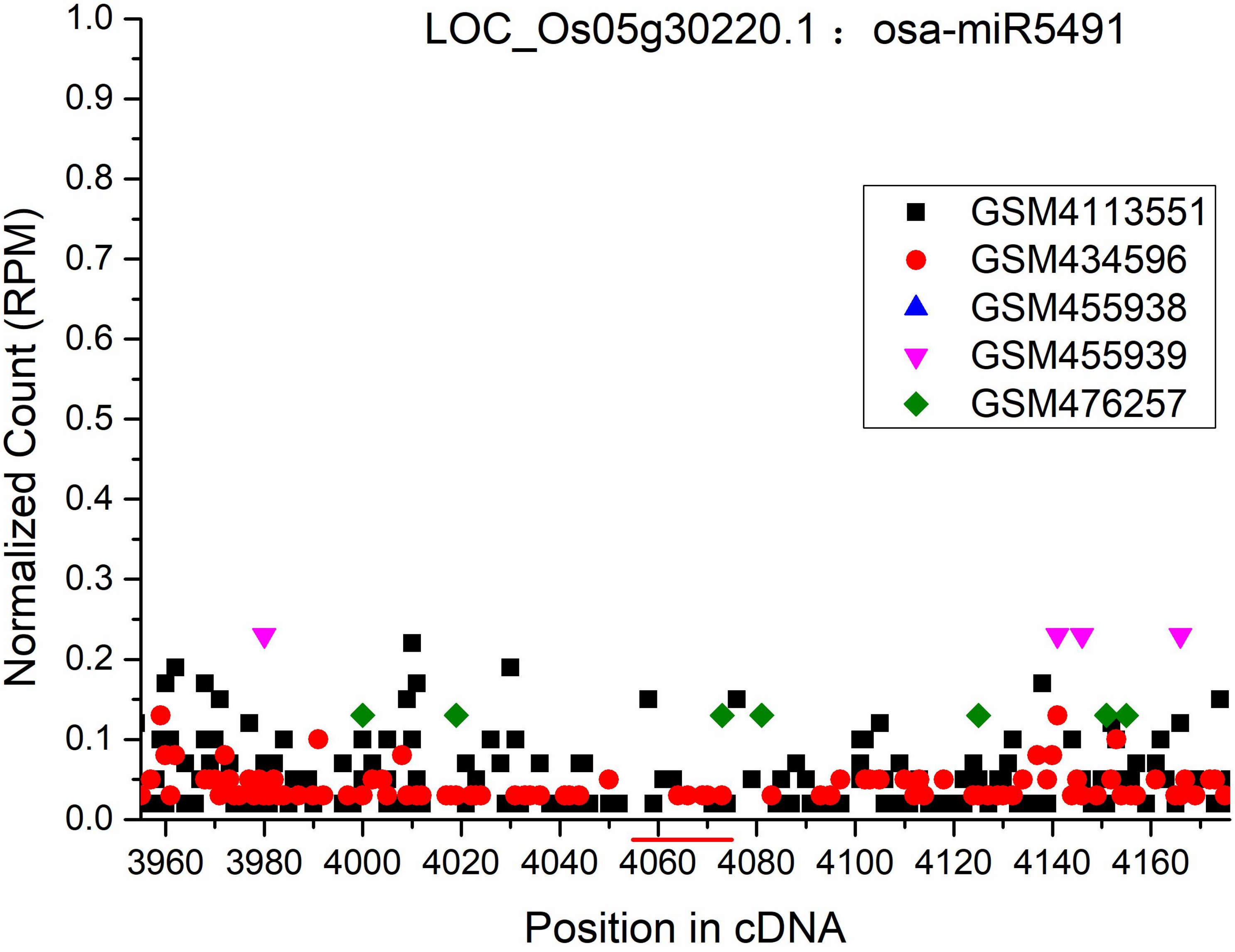

LOC_Os05g30220.1 ： osa-miR5491
Normalized Count (RPM)
1.0
0.9
0.8
0.7
0.6
0.5
0.4
0.3
0.2
0.1
0.0
GSM4113551
GSM434596
GSM455938
GSM455939
GSM476257
3960
3980
4000
4020
4040
4060
4080
4100
4120
4140
4160
Position in cDNA

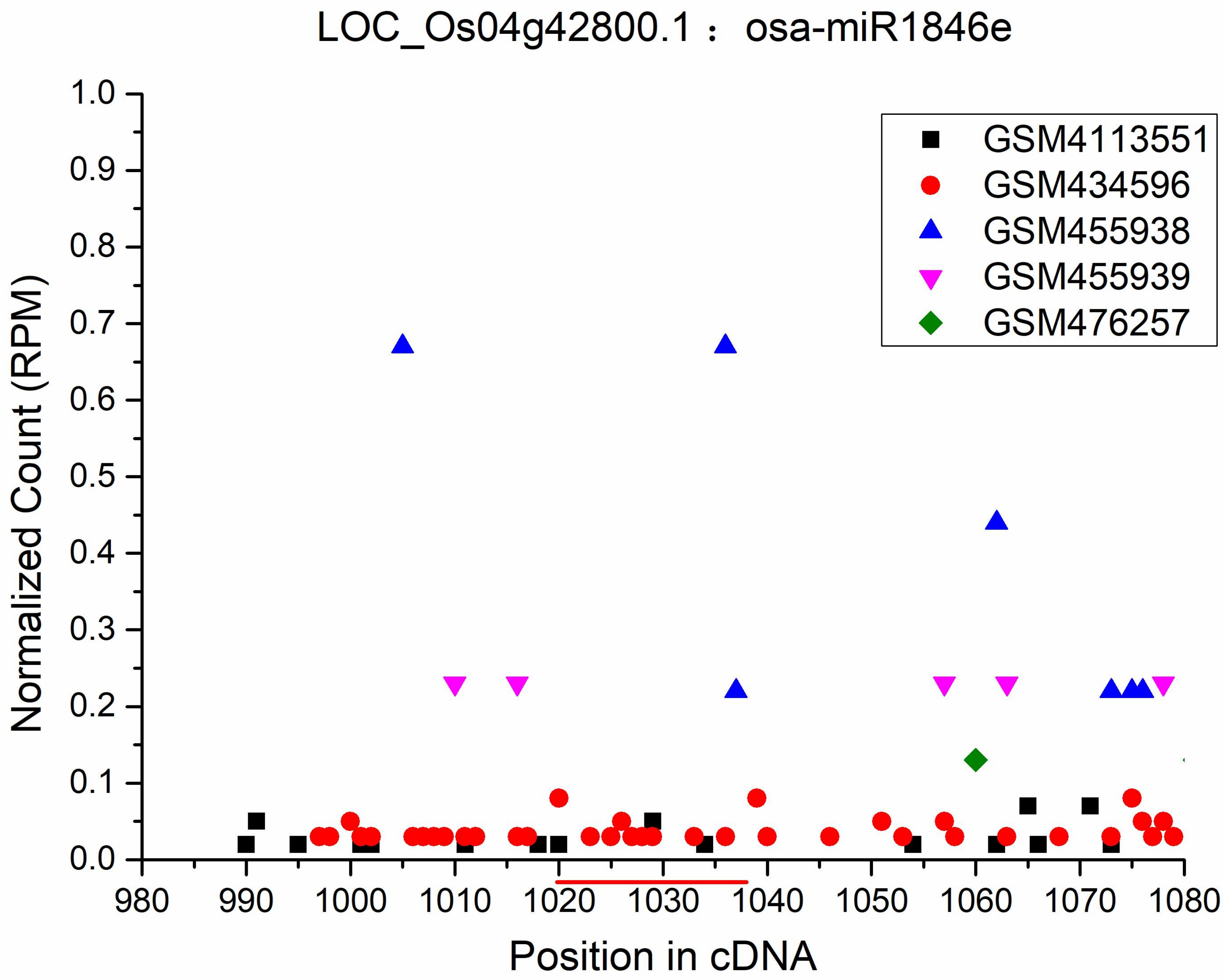
LOC_Os04g42800.1：osa-miR1846e
Normalized Count (RPM)
Position in cDNA
GSM4113551
GSM434596
GSM455938
GSM455939
GSM476257
0.0
0.1
0.2
0.3
0.4
0.5
0.6
0.7
0.8
0.9
1.0
980
990
1000
1010
1020
1030
1040
1050
1060
1070
1080

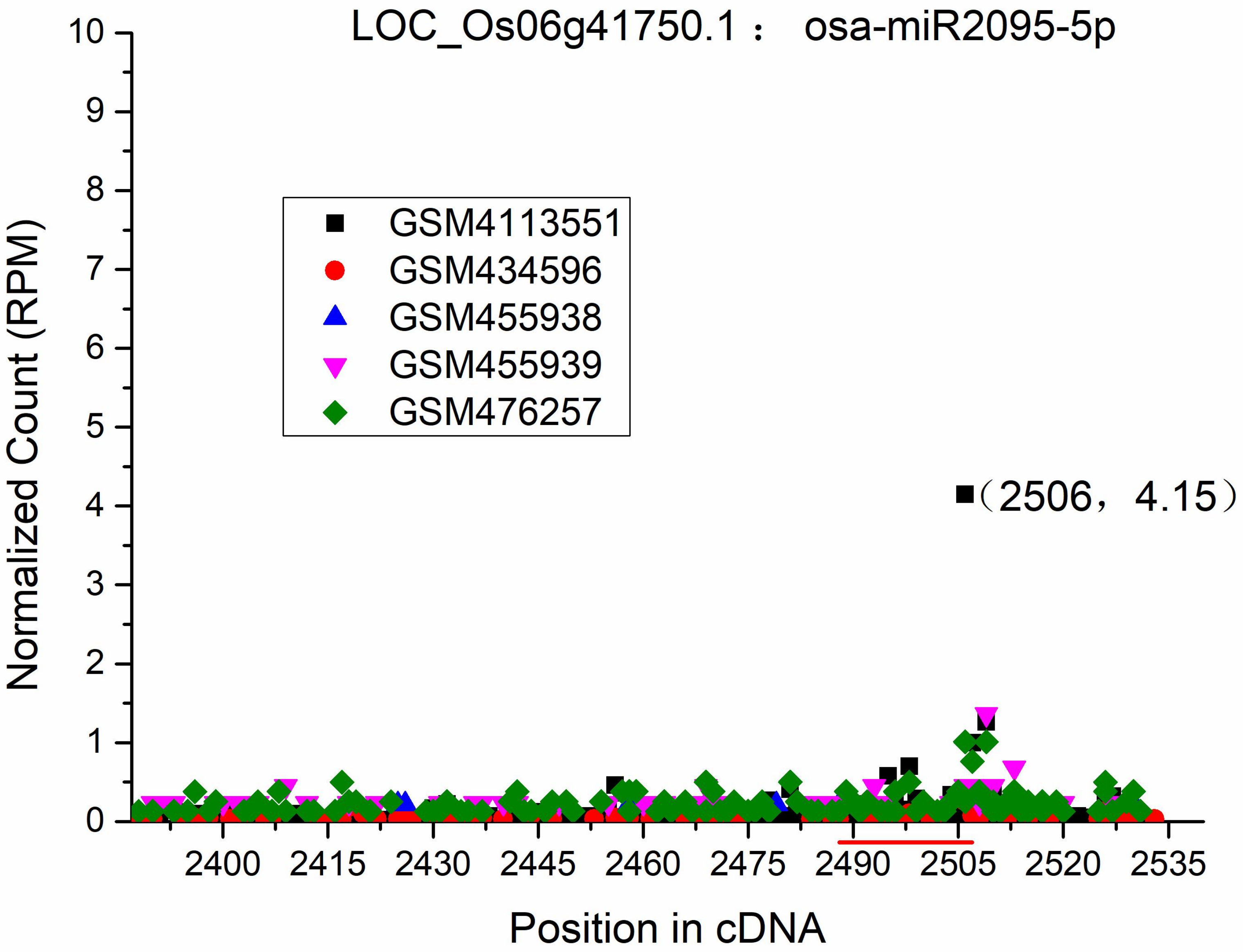
LOC_Os06g41750.1： osa-miR2095-5p
Normalized Count (RPM)
0
1
2
3
4
5
6
7
8
9
10
GSM4113551
GSM434596
GSM455938
GSM455939
GSM476257
（2506，4.15）
2400
2415
2430
2445
2460
2475
2490
2505
2520
2535
Position in cDNA
